# Perivascular Adipose Tissue's Impact on Norepinephrine-Induced Contraction of Mesenteric Resistance Arteries

**DOI:** 10.3389/fphys.2017.00037

**Published:** 2017-02-08

**Authors:** Nadia Ayala-Lopez, Janice M. Thompson, Stephanie W. Watts

**Affiliations:** Department of Pharmacology and Toxicology, Michigan State UniversityEast Lansing, MI, USA

**Keywords:** norepinephrine, monoamine oxidase, semicarbazide sensitive amine oxidase, amine oxidase (copper-containing), isometric contraction, uptake transporters, norepinephrine transporter, organic cation transporter

## Abstract

**Background:** Perivascular adipose tissue (PVAT) can decrease vascular contraction to NE. We tested the hypothesis that metabolism and/or uptake of vasoactive amines by mesenteric PVAT (MPVAT) could affect NE-induced contraction of the mesenteric resistance arteries.

**Methods:** Mesenteric resistance vessels (MRV) and MPVAT from male Sprague-Dawley rats were used. RT-PCR and Western blots were performed to detect amine metabolizing enzymes. The Amplex® Red Assay was used to quantify oxidase activity by detecting the oxidase reaction product H_2_O_2_ and the contribution of PVAT on the mesenteric arteries' contraction to NE was measured by myography.

**Results:** Semicarbazide sensitive amine oxidase (SSAO) and monoamine oxidase A (MAO-A) were detected in MRV and MPVAT by Western blot. Addition of the amine oxidase substrates tyramine or benzylamine (1 mM) resulted in higher amine oxidase activity in the MRV, MPVAT, MPVAT's adipocyte fraction (AF), and the stromal vascular fraction (SVF). Inhibiting SSAO with semicarbazide (1 mM) decreased amine oxidase activity in the MPVAT and AF. Benzylamine-driven, but not tyramine-driven, oxidase activity in the MRV was reduced by semicarbazide. By contrast, no reduction in oxidase activity in all sample types was observed with use of the monoamine oxidase inhibitors clorgyline (1 μM) or pargyline (1 μM). Inhibition of MAO-A/B or SSAO individually did not alter contraction to NE. However, inhibition of both MAO and SSAO increased the potency of NE at mesenteric arteries with PVAT. Addition of MAO and SSAO inhibitors along with the H_2_O_2_ scavenger catalase reduced PVAT's anti-contractile effect to NE. Inhibition of the norepinephrine transporter (NET) with nisoxetine also reduced PVAT's anti-contractile effect to NE.

**Conclusions:** PVAT's uptake and metabolism of NE may contribute to the anti-contractile effect of PVAT. MPVAT and adipocytes within MPVAT are a source of SSAO.

## Introduction

Perivascular adipose tissue (PVAT) makes up the fourth layer of most blood vessels (Chaldakov et al., 2007). PVAT is appreciated for its effects on the vasculature, from mediating relaxation (Brandes, 2007; Fesus et al., 2007) to stimulating contraction of arteries by the release of catecholamines (Gao et al., 2006; Ayala-Lopez et al., 2014). Increased visceral fat, which includes mesenteric fat, is associated with increased cardiovascular risk (Kotchen, 2010). Thus, our focus is on the mesenteric PVAT because it surrounds resistance arteries, which provide increased vascular resistance in hypertension (for a review: Intengan and Schiffrin, 2000). Investigating mesenteric PVAT's mechanisms of handling NE, the sympathetic neurotransmitter, is relevant in understand the pathology in this disease because sympathetic nervous system activity is increased in obesity-related hypertension (Hall et al., 2010).

In normal (non-disease) animal models, PVAT inhibits contraction of the underlying arteries to several agonists, including norepinephrine (NE) (Soltis and Cassis, 1991; Löhn et al., 2002) in a mechanism dependent on transport-dependent uptake of NE. Mesenteric and aortic PVAT can take up NE applied exogenously, which could contribute to PVAT's anti-contractile effect on mesenteric and aortic arteries exposed to NE (Ayala-Lopez et al., 2015). Nerve terminals terminate NE activation of post-synaptic receptors through uptake of NE followed by storage into vesicles and/or by degradation by NE metabolizing enzymes. Like nerves, PVAT also has a storage of catecholamines (Ayala-Lopez et al., 2014) but whether degradation of NE occurs within PVAT has not been investigated.

PVAT affects arterial contraction through an adrenergic system that is complete with the release and uptake of NE (Ayala-Lopez et al., 2014, 2015). We hypothesize that PVAT's anti-contractile effect is through its amine metabolizing activity in addition to NE uptake. The NE metabolizing enzymes monoamine oxidases A (MAO-A) and monoamine oxidase B (MAO-B) are present in human white adipose tissue adipocytes isolated from mammary and abdominal lipectomies (Pizzinat et al., 1999). One study supports the presence in adipose tissue (canine subcutaneous) of catechol-o-methyl transferase (COMT), another metabolizer of NE (Belfrage et al., 1977). Lastly, the dual function enzyme, semicarbazide sensitive amine oxidase (SSAO), also known as vascular adhesion protein-1 (VAP-1) for its role as a leukocyte adhesion molecule, catalyzes the deamination of amines. SSAO is shed from the plasma membrane of adipocytes. Circulating levels of SSAO are elevated in cardiovascular disease (Abella et al., 2004) and atherosclerosis (Karadi et al., 2002) making SSAO in PVAT an interesting target of study. NE is a substrate for SSAO in rat brown adipose tissue (Barrand and Callingham, 1982). The presence of and the effect that amine metabolism in PVAT on vascular contraction to NE is not known.

To study NE handling in PVAT, we used mesenteric resistance arteries with and without PVAT. Additionally, PVAT was separated into its fractional components, the adipocyte fraction (AF) and the stromal vascular fraction (SVF). The stromal vascular fraction is the non-buoyant fraction separated from PVAT. It contains fibroblasts, mesenchymal stem cells, lymphocytes, macrophages and endothelial cells (Szasz et al., 2013). Gene expression analysis and Western blots helped to identify which NE metabolizing enzymes are present in mesenteric resistance vessels (MRV- pooled mesenteric resistance arteries and veins) and in their surrounding MPVAT. We measured oxidase activity after the addition of tyramine or benzylamine, substrates of MAO and SSAO (Visentin et al., 2005) and used pharmacological inhibitors of amine oxidases to determine which oxidases are important for the activity observed. Contraction to NE was recorded in isolated third-order mesenteric arteries with and without PVAT exposed to inhibitors of MAO-A/B, SSAO and NE transporters. Our work presents insight into the impact of PVAT on arterial contraction to NE.

## Materials and methods

### Chemicals

Norepinephrine hydrochloride, nisoxetine hydrochloride, semicarbazide hydrochloride, and catalase were purchased from Sigma-Aldrich (St. Louis, MO). Pargyline hydrochloride for the contractility experiments was purchased from Cayman Chemical (Ann Arbor, MI). Corticosterone was purchased from Tocris Bioscience (United Kingdom). Pargyline and clorgyline used in the oxidase assay experiments were supplied within the Amplex® Red Monoamine Oxidase Assay Kit (cat# A12214, ThermoFisher Scientific, Grand Island, NY USA).

### Animals

Male Sprague-Dawley rats (225–275 g or ~8–10 weeks of age, Charles River, Indianapolis, IN USA) were used. All protocols were approved by the MSU Institutional Animal Care and Use Committee and follow the “Guide for the Care and Use of Laboratory Animals,” 8th edition, 2011. Rats were anesthetized with sodium pentobarbital (60–80 mg/kg, IP). Anesthesia was verified by lack of paw pinch and eye blink reflexes. Death was assured by pneumothorax and exsanguination after which tissues were removed for one of the following protocols.

### Tissue dissection

The liver was collected, snap frozen in liquid N_2_ and saved to serve as a positive control for the RT-PCR and Western blot assays. Mesentery, brain and aorta were collected in physiological salt solution (PSS); in mM; 130 NaCl; 4.7 KCl; 1.18 KH_2_PO_4_; 1.17 MgSO_4_ 7H_2_O; 14.8 NaHCO_3_; 5.5 dextrose; 0.03 CaNa_2_ ethylenediaminetetraacetic acid; 1.6 CaCl_2_ (pH 7.2). A portion the rat brain corresponding to the midbrain and pons was dissected and the aorta was cleaned of PVAT. Both samples were then saved in potassium phosphate buffer (50 mM) for the oxidase activity assay. The same tissues (brain and aorta without PVAT) from a separate set of animals were dissected, frozen in liquid N_2_ and saved for RT-PCR. MPVAT and the associated mesenteric resistance arteries and veins (MRV) were separated out in a Sylgard® coated petri dish in PSS with the aid of a stereomicroscope. The MPVAT was either used for fractionation, frozen in liquid N_2_ for protein isolation, or saved in potassium phosphate buffer (50 mM) for the oxidase assay. The MRV were either frozen in liquid N_2_ for protein or RNA isolation, or saved in potassium phosphate buffer (50 mM). Samples for the oxidase activity assays were saved at −20°C and used within 1 week. Mesenteric resistance arteries (2 mm in length) with or without PVAT for the use in isometric contraction experiments were dissected out in a Sylgard® coated petri dish in PSS with the aid of a stereomicroscope.

### Adipocyte and SVF isolation

MPVAT was added to 1 mL of PSS with 1 mg/mL collagenase from *Clostridium histolyticum* type IA (cat# C9891, Sigma) and incubated at 37°C with gentle agitation until fully digested. The sample was centrifuged at 200 × g for 5 min after which the SVF was transferred into a separate tube. The fractions were then washed six times by adding 1 mL of PSS and centrifuging at 200 × g for 10 min. This protocol is one used to routinely isolate adipocytes and that has been verified by other groups to satisfactorily exclude other cell types (Vargovic et al., 2013). Purity of the isolation (>95% adipocytes) was verified by counting the adipocytes vs. non-adipocytes present with a hemocytometer. Phase contrast images of the fractions were taken with a 20 × objective (Hi PLAN I 20X/ 0.30 PH1) on an inverted microscope DMi1 (Leica, Buffalo Grove, IL, USA) using Leica Application Suite (LAS). The PSS was then removed and the samples were placed in 50 mM potassium phosphate buffer to be used in the oxidase assay before freezing or snap frozen in liquid N_2_ for protein isolation.

### Real-time PCR

All tissues (brain, liver, MRV, MPVAT, and aorta) were homogenized using the Bead Ruptor 24 (Omni International, NW Kennesaw, GA). RNA was extracted with the Quick RNA MiniPrep kit (cat# R1054, Zymo Research Corporation, Irving, CA USA) and purity (260/280 and 260/230 ratios ≥ 1.8) was verified using a Nanodrop 2000C spectrophotometer (Thermo Scientific, Wilmington, DE USA). The mRNA (1 μg) was reverse transcribed with the High-Capacity cDNA Reverse Transcription Kit (cat# 4368814, ThermoFisher Scientific). RT-PCR was performed using PerfeCTa FastMix II, ROX (cat# 95119, Quanta Biosciences, Gaithersburg, MD USA) on the ABI 7500 Fast Real Time PCR system (Life Technologies, Carlsbad, CA USA) with the following parameters: 95°C for 20 s, 95°C for 1 s and 60°C for 20 s for 40 cycles. Taqman Primers were purchased from ThermoFisher Scientific. The sequences are proprietary. Thus, we have listed the catalog numbers which are as follows: *Aoc3* (cat# 4448892, assay ID: Rn01452826_m1), *Comt* (cat#4448892, assay ID: Rn01404927_g1: *Actb* (cat#4448892, assay ID: Rn00667869_m1), *Maoa* (cat#4448892, assay ID: Rn01430950_m1), *Maob* (cat#4448892, assay ID: Rn00566203_m1). Measures were normalized to β-actin (*Actb*) and expressed as fold change relative to the positive control tissue as described by Livak and Schmittgen (2001).

### Western blots

MPVAT and MRV protein was isolated in phosphate buffered saline (PBS) with protease inhibitors [sodium orthovanadate (1 mM), aprotinin/leupeptin (100 μg/ml) and phenylmethylsulfonyl fluoride (1 mM)] and homogenized using the Bead Ruptor 24 (Omni International). The protein from the positive controls was isolated as follows. Stomach fundus and gut mucosa were dissected from the rat and were placed into RIPA buffer (cat# R3792, Teknova, Hollister, CA) with the above protease inhibitors before homogenizing with the Bead Ruptor 24 (Omni International) and centrifuging at 10,000 rpm for 10 min. Protein from the aorta was isolated in 1X lysis buffer [Tris HCl (62.5 mM) pH 7.8, 2% SDS, 10% glycerol] with the above protease inhibitors, frozen in liquid N_2_, homogenized with mortar and pestle followed by a centrifugation at 11,000 rpm for 10 min. Supernatants were then separated. The Jurkat whole cell lysate was purchased from Santa Cruz Biotechnology (cat# SC-2204, Dallas, TX). The protein concentrations were determined using the Bicinchoninic Acid Protein Assay Kit (cat# BCA1, Sigma-Aldrich). The protein samples (50 μg) were separated on 10% SDS polyacrylamide gels using the Bio-Rad Mini Protean 3 system. Protein was transferred to PVDF-FL (MAO-A) or nitrocellulose (MAO-B, COMT, VAP-1) and blocked for 3 h at 4°C in 4% chicken egg ovalbumin (MAO-A, β-actin), LI-COR Odyssey Blocking Buffer (MAO-B, COMT) or 5% bovine serum albumin (VAP-1). Primary antibody [MAO-A, 1:200 (epitope corresponds to amino acids 458–527 of MAO-A of human origin; Santa Cruz Biotechnology, cat#SC-20156); MAO-B, 1:200 (epitope near the C-terminus of MAO-B of human origin; Santa Cruz Biotechnology, cat#SC-18401); COMT, 1:200 (epitope corresponding to amino acids 1-271 of COMT of human origin; Santa Cruz Biotechnology, cat#SC-25844); VAP1, 1:200 (epitope near the C-terminus of VAP-1 of human origin; Santa Cruz Biotechnology, cat#SC-13741); or β-actin, 1:2000 (Sigma, cat#A3854)] was incubated overnight in blocking buffer at 4°C. Antibody was recovered and blots were washed with TBS + 0.1% Tween 20 (TBS-T) for 10 min at 4°C (three times). Blots were then incubated with species-specific LI-COR IRDye 800 secondary antibody (1:1000, MAO-A, MAO-B, COMT, VAP1) or LI-COR IRDye 700 secondary antibody (1:1000, β-actin) in LI-COR Odyssey Blocking Buffer for 1 h at 4°C, followed by washes with TBS-T for 10 min at 4°C (three times). Experiments using a competing peptide (MAO-B: cat#SC-18401 P, VAP-1: SC-13741 P, Santa Cruz Biotechnology) were performed as above except for that before the primary antibody was added to the blot it was incubated overnight at 4°C with the competing peptide at 5x the concentration of the antibody. Bands were visualized using the LI-COR Odyssey Classic or the LI-COR Odyssey CLx. Densitometry was completed with Image J.

### Oxidase activity assay

Samples were homogenized with the Bead Ruptor 24 (Omni International). The samples were centrifuged at 600 × g for 10 min and the supernatant transferred to new tubes. The protein concentration of an aliquot of the sample was measured before the oxidase assay using a Bicinchoninic Acid Protein Assay Kit (cat# BCA1, Sigma-Aldrich) to inform us on the volume of the sample to load into the assay. MPVAT and the adipocyte fraction (AF) samples were loaded into a black clear-bottom 96-well plate at 20 mg of protein per well. Twice this protein, 40 mg, was loaded for the MRV, the SVF, brain (positive control for MAO) and aorta (positive control for SSAO). More protein was used for non-adipocyte containing tissues because the baseline H_2_O_2_ production in these extracts was less. Thus, more was loaded to ensure H_2_O_2_ would be detected. Samples in 1X reaction buffer were added into each well of a 96-well plate with an inhibitor of metabolism/or vehicle. The plate was incubated at room temperature for 30 min. Then, the substrate was added (either tyramine or benzylamine; 1 mM), with 100 μL of the Amplex® Red Reagent to start the reaction. The plate was incubated for another 30 min at room temperature after which the fluorescence was read at 530–560 nm excitation and 590 nm emission on the Infinite M1000 PRO (Tecan Group Ltd, Männedorf, Switzerland). Measures were compared to a resorufin standard curve and expressed as pmol/min/mg of protein.

### Isometric contraction

Rat third-order mesenteric resistance arteries cleaned of fat (−PVAT) or with fat intact (+PVAT) were mounted into a Multi Wire Myograph System 620M (Danish Myo Technology, Denmark). Data were acquired using a PowerLab Data Acquisitions unit (ADInstruments, Colorado Springs, CO, USA). Baths contained warmed, oxygenated PSS. Rings were pulled to optimum resting tension (13.3 kpa) with the aid of the DMT Normalization Module for LabChart software and PowerLab (Danish Myo Technology and ADInstruments; http://cdn.adinstruments.com/adi-web/brochures/DMT-Normalization-2011.pdf) and equilibrated for 1 h with washes every 20 min. The arteries were exposed to an initial concentration of 60 mM KCl to test viability. Tissues were washed and tone returned to baseline and exposed to another concentration of 60 mM KCl to elicit the maximum contraction. The 60 mM KCl maximum contraction was what we normalized the force of contraction to. Tissues were then washed and returned to baseline. Either vehicle or inhibitor was added for 1 h without washing. NE was then added in a cumulative fashion, with significant time necessary for a response to plateau prior to the next addition. If no response was recorded within 3 min, the next concentration of NE was applied. Tissues were washed and a final 60 mM KCl addition was performed to test for tissue viability at the end of the experiment. Data were expressed as percent maximum contraction. The final contraction to 60 mM KCl was compared to the initial contraction (Data Supplementary Figures 4, 5). If the tissue did not contract to the final concentration of 60 mM KCl, the data for that experiment was not used.

### Statistical analysis

Data are reported as means ± SEM for number of animals indicated by N or near each bar within the graphs. Statistical analyses were performed with GraphPad Prism 6.0 (GraphPad Software, Inc., La Jolla, CA). Outliers were identified and removed following a Grubb's test. Contraction was reported as means ± SEM as a percentage of the initial contraction to 60 mM KCl. Potency means (−logEC_50_, M) were calculated using GraphPad Prism 6.0. Where a maximum was not achieved, the values are estimated and true potencies equal or greater than that reported. *P* < 0.05 was considered statistically significant.

## Results

### *Aoc3* (the gene for SSAO) and *Maoa* (the gene for MAO-A) are highly expressed in MPVAT

**Figure 1:**
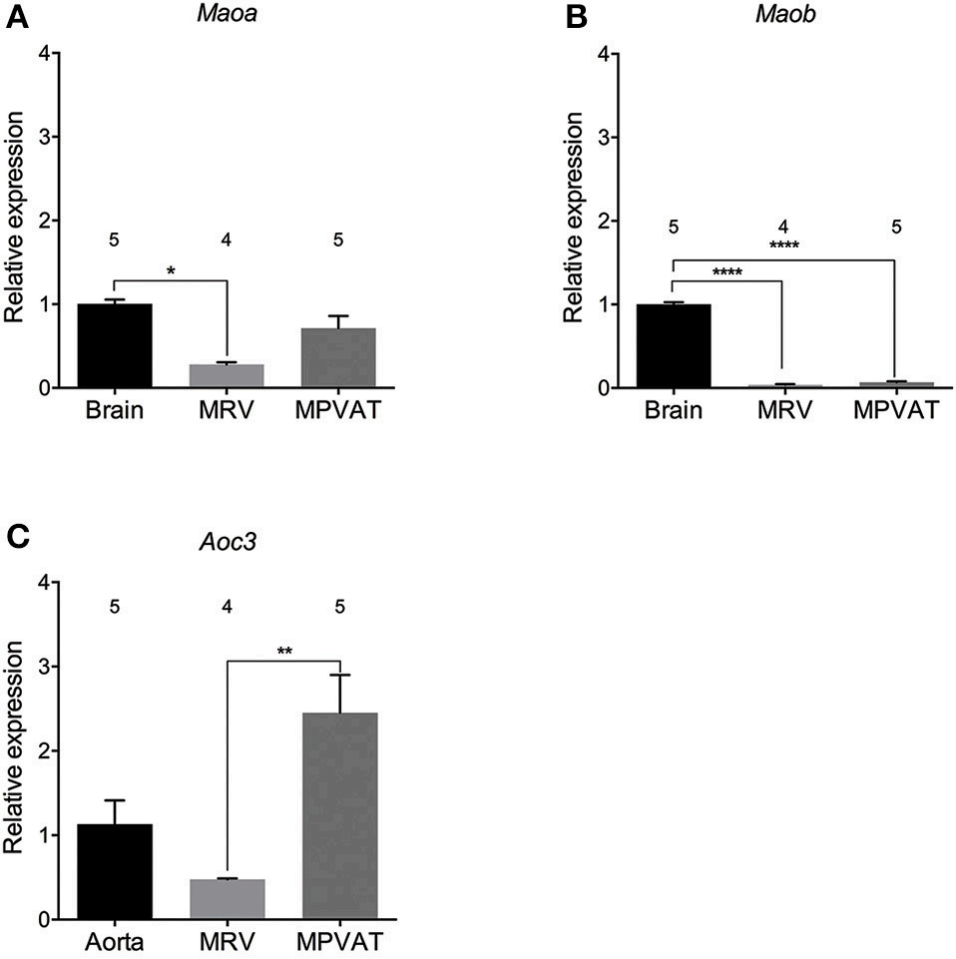
*****Maoa*** and ***Aoc3*** are highly expressed in rat mesenteric PVAT**. Amine oxidase gene expression of **(A)** *Maoa*, **(B)** *Maob*, and **(C)** *Aoc3* (gene for SSAO) in mesenteric resistance vessels (MRV) and in mesenteric PVAT (MPVAT) normalized to β-actin as the reference gene. Bars represent means ± SEM. Means were compared with a one-way ANOVA followed by the Holm-Sidak's multiple comparisons test for parametric data sets (*Maob*) or the Kruskal-Wallis followed by the Dunn's test for multiple comparisons for the non-parametric data sets (*Maoa* and *Aoc3*). ^*^p < 0.05, ^**^p < 0.01, ^****^p < 0.0001. The number above each bar indicates the number of animals used.

MPVAT and the underlying artery-vein pair (Data Supplementary Figure 1) were dissected from male Sprague-Dawley rats and analyzed for the expression of amine metabolizing enzyme genes. Relative *Maoa* expression in the MPVAT was similar to that in the brain (Figure 1A), the positive control for *Maoa* and *Maob* (Jahng et al., 1997). However, MRV expression of *Maoa* was significantly lower than in the brain but not the MPVAT. *Maob* expression was significantly lower in both the MRV and in the MPVAT vs. the brain positive control (Figure 1B). When compared to the liver, a positive control for *Comt* expression (Karhunen et al., 1994), MRV and MPVAT had low levels of *Comt* expression (less than 0.01-relative expression from liver for both; data not shown). By contrast, *Aoc3* (amine oxidase, copper containing 3; the gene for SSAO) was expressed at higher levels in the MPVAT (Figure 1C) vs. the MRV when compared relative to the aorta, a positive control for *Aoc3* expression (Wanecq et al., 2006).

### MAO-A and SSAO protein is present in MRV and MPVAT

**Figure 2:**
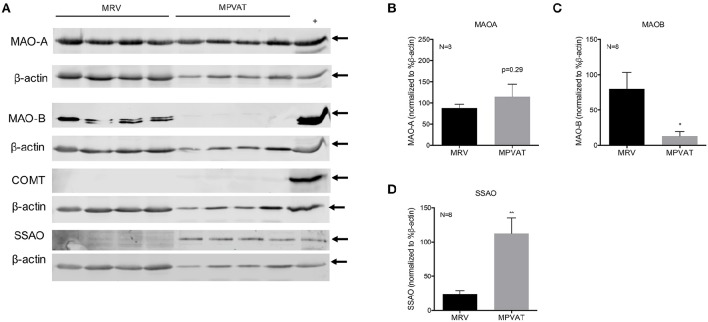
**MAO-A, MAO-B, and SSAO but not COMT are present in rat MRV and MPVAT. (A)** Western blot analysis of monoamine metabolism enzymes MAO-A, MAO-B, COMT, and SSAO in mesenteric resistance vessels (MRV) and in mesenteric PVAT (MPVAT) from eight animals. Positive controls were gut mucosa for MAO-A, stomach fundus for MAO-B, Jurkat cells for COMT and aorta for SSAO. Densitometry analysis of Western blot bands for **(B)** MAO-A, **(C)** MAO-B, **(D)** SSAO. Bars represent the means ± SEM. Western blot densitometry was statistically analyzed with a paired Student's *t*-test. ^*^p < 0.05, ^**^p < 0.01.

Western blot analysis of protein isolated from the MRV and associated MPVAT, revealed presence of MAO-A, MAO-B and SSAO (Figure 2A). Consistent with there being no difference between the mRNA expression for *Maoa* in the MRV and MPVAT, there was no statistical difference in relative protein signal between the MRV and MPVAT (*p* = 0.29; Figures 2A,B). MAO-B signal was lower the MPVAT than in the MRV (Figures 2A,C). Bands for COMT were not detected in either the MRV or the MPVAT (Figure 2A; densitometry not shown). SSAO signal was higher in the MPVAT vs. the MRV (Figures 2A,D), also consistent with Figure 1C.

### SSAO mediates tyramine and benzylamine-induced amine oxidase activity in MPVAT

**Figure 3:**
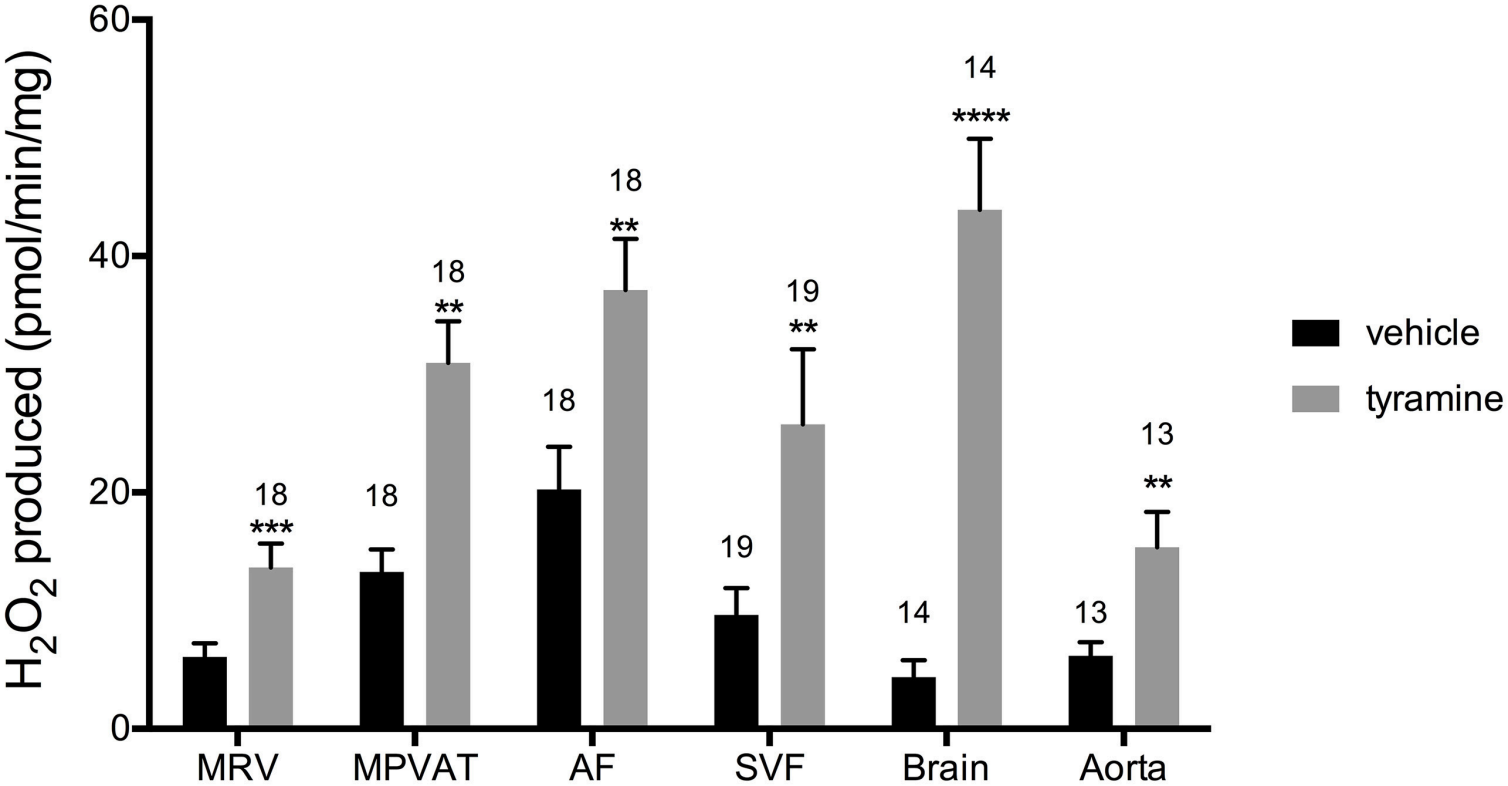
**Tyramine drives amine oxidase activity in the MRV, MPVAT, AF, and SVF**. Tyramine, a substrate for amine oxidases, increased the oxidase activity in the mesenteric resistance vessels (MRV), mesenteric PVAT (MPVAT), adipocyte fraction (AF), and the stromal-vascular fraction (SVF). The brain and aorta were also tested as positive controls. Bars represent the means ± SEM for the number of animals indicated above each bar. Means were compared by a Mann-Whitney test. ^**^p < 0.01, ^***^p < 0.001, ^****^p < 0.0001 vs. control of that same sample type.

Oxidation of amines in the MRV and in the MPVAT could alter vascular tone through the removal of vasoactive amines and through the release of the reaction product H_2_O_2_. To quantify the oxidase activity in these tissues, the MRV and MPVAT were carefully separated. MPVAT was split into its constituent fractions, the AF and the SVF. A microscopic image of the separated fractions is shown in Data Supplementary Figure 2, which represents the AF and SVF used in this study. Tyramine, a substrate for MAO-A, MAO-B and SSAO, was added to the sample extracts to drive oxidase activity. Addition of tyramine increased the oxidase activity (H_2_O_2_ produced) in the MRV, MPVAT, the AF, and the SVF vs. vehicle (H_2_O) (Figure 3). The brain contains both MAO-A and B activity but low SSAO activity (Kalaria et al., 1988; Castillo et al., 1999). Thus, the brain served as a positive control for the MAOs. The brain had higher oxidase activity in response to tyramine vs. vehicle. The aorta, the positive control for SSAO, also had higher activity to tyramine vs. vehicle.

To determine which amine oxidase(s) contributed to the tyramine-driven H_2_O_2_ production in the mesentery, the MRV, MPVAT, AF and the SVF were incubated with inhibitors to each of the amine oxidases (MAO-A, MAO-B, and SSAO) before adding tyramine. Clorgyline, an irreversible inhibitor of MAO-A, was used due to its specificity to MAO-A (IC_50_ of 0.03 μM and 8 μM at MAO-B (Ozaita et al., 1997) and its lack of inhibition of SSAO (Clarke et al., 1982). Pargyline (1 μM) was used to specifically inhibit MAO-B (K_i_ 0.5 μM) and a higher concentration (10 μM) was used to inhibit both MAO-A and B (K_i_ for pargyline at MAO-A = 15 μM Fowler et al., 1982). Semicarbazide was used to specifically inhibit SSAO, at a concentration (1 mM) routinely used in monoamine assays to selectively inhibit SSAO activity (K_i_ 15 μM Lizcano et al., 1996; Repessé et al., 2015). Oxidase activity was quantified as the amount of H_2_O_2_ produced per minute normalized to the amount of protein.

**Figure 4:**
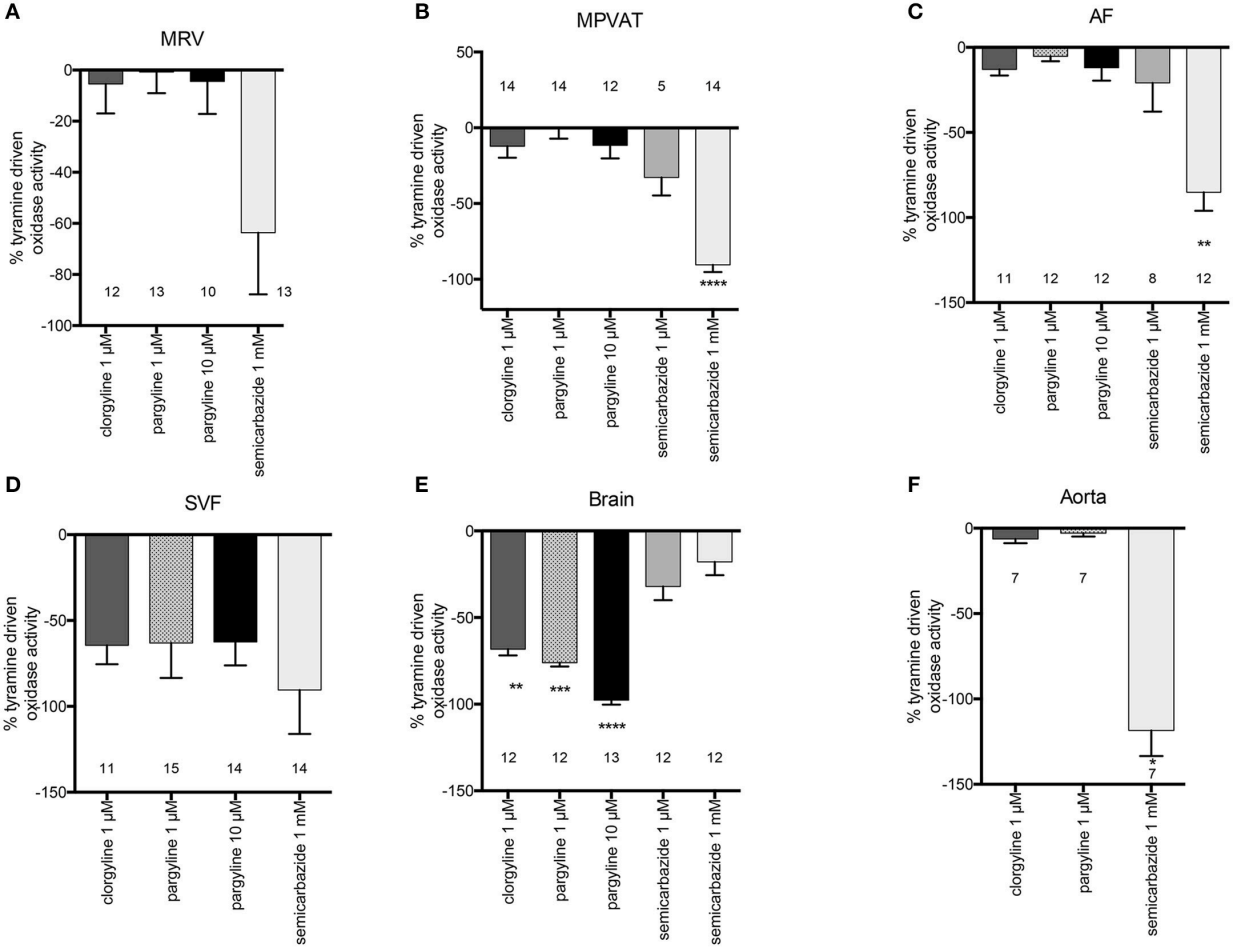
**SSAO mediates tyramine-driven amine oxidase activity in the MPVAT and its fractions, the AF and SVF, but not the MRV**. Percent inhibition of tyramine driven oxidase activity by pharmacological inhibitors of MAO-A (1 μM clorgyline), MAO-B (1 μM pargyline), both MAO-A and MAO-B (10 μM pargyline) or SSAO (1 mM semicarbazide) in the **(A)** mesenteric resistance vessels (MRV), **(B)** mesenteric PVAT (MPVAT), **(C)** adipocyte fraction (AF), **(D)** stromal vascular fraction (SVF), and the positive controls **(E)** brain (positive control for MAO-A/B), and **(F)** aorta (positive control for SSAO). Bars represent the means ± SEM for the number of animals indicated near each bar. Means were analyzed by a Kruskal-Wallis test followed by the Dunn's test for multiple comparisons. ^*^p < 0.05, ^**^p < 0.01, ^***^p < 0.001, ^****^p < 0.0001 vs. vehicle control (no inhibition).

Tyramine-driven oxidase activity in the MRV was not reduced by the addition of clorgyline or pargyline (Figure 4A). However, there was a non-statistically significant reduction with 1 mM semicarbazide (Figure 4A). On the other hand, semicarbazide significantly reduced (almost by 100%) the tyramine-driven oxidase activity in the MPVAT and the AF (Figures 4B,C).

Oxidase activity in the SVF was not significantly reduced by inhibition of the MAOs or SSAO vs. vehicle (Figure 4D). However, each inhibitor (clorgyline, pargyline, and semicarbazide) caused large non-significant reductions in oxidase activity. No one enzyme had activity that was higher over the other enzymes. Thus, each amine oxidase (MAO-A, MAO-B, and SSAO) may contribute to oxidase activity in the SVF. The brain had reduced oxidase activity to clorgyline (1 μM- specific for MAO-A), pargyline (1 μM- specific for MAO and 10 μM- inhibits both MAO-A and MAO-B), but no reduction with the SSAO inhibitor semicarbazide (Figure 4E). Oxidase activity in the aorta was abolished by semicarbazide (1 mM); by contrast, there was not reduction in oxidase activity by clorgyline (1 μM) or pargyline (1 μM) (Figure 4F). The results for these controls support that the assay can detect and distinguish between SSAO and MAO activity.

**Figure 5:**
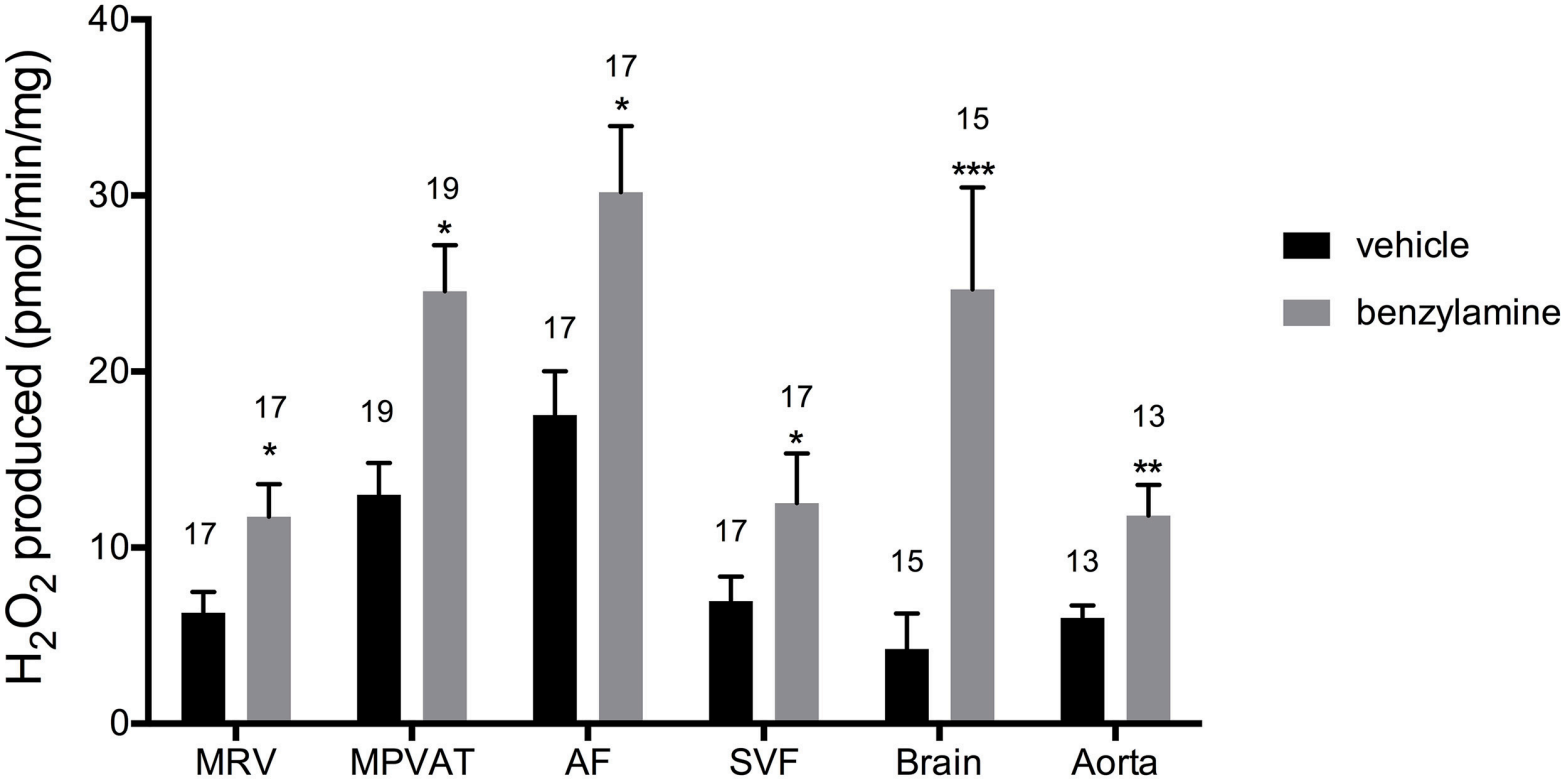
**Benzylamine drives amine oxidase activity in MRV, MPVAT, AF, and the SVF**. MPVAT, mesenteric PVAT; AF, adipocyte fraction; MRV, mesenteric resistance vessels; SVF, stromal vascular fraction. The brain and aorta were also tested as positive controls. Bars represent the means ± SEM for the number of animals indicated above each bar. Means were compared by a two-way ANOVA followed by a Mann-Whitney or a *t*-test. ^*^p < 0.05, ^**^p < 0.01, ^***^p < 0.001 vs. control of that same sample type.

**Figure 6:**
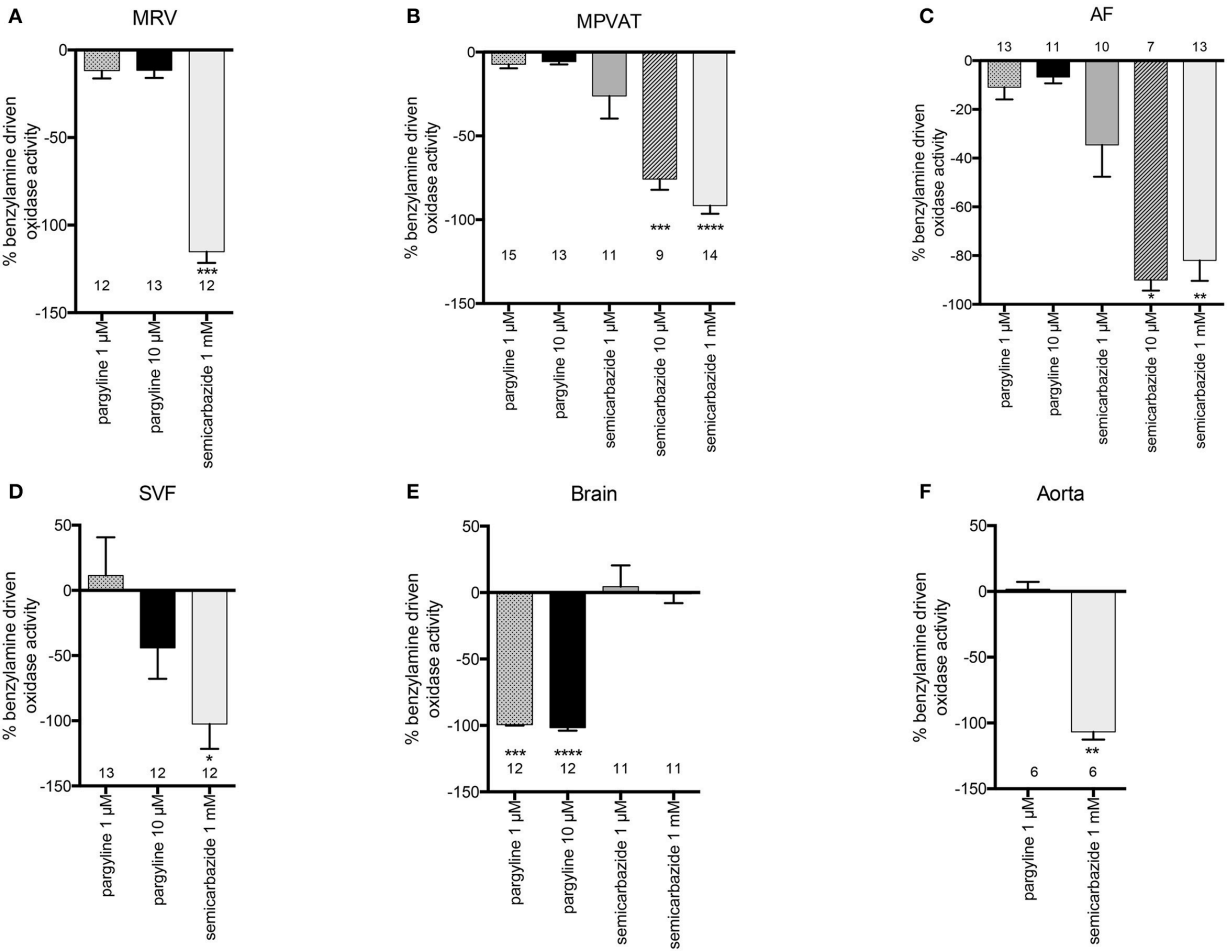
**SSAO mediates benzylamine-driven amine oxidase activity in the MRV, MPVAT and its fractions, the AF and SVF**. Percent inhibition of benzylamine driven oxidase activity by pharmacological inhibitors of MAO-B (1 μM pargyline), both MAO-A and MAO-B (10 μM pargyline) or SSAO (1 mM semicarbazide) in the **(A)** mesenteric resistance vessels (MRV), **(B)** mesenteric PVAT (MPVAT), **(C)** adipocyte fraction (AF), **(D)** stromal vascular fraction (SVF), and the positive controls **(E)** brain (positive control for MAO-A/B), and **(F)** aorta (positive control for SSAO). Bars represent the means ± SEM for the number of animals indicated above each bar. Means were analyzed by a Kruskal-Wallis test followed by the Dunn's test for multiple comparisons. ^*^p < 0.05, ^**^p < 0.01, ^***^p < 0.001, ^****^p < 0.0001 vs. vehicle control (no inhibition).

Benzylamine differs from tyramine in that it is a substrate for MAO-B and SSAO only (and not MAO-A). Addition of benzylamine increased oxidase activity vs. vehicle in the MPVAT, AF, SVF, brain, and aorta (Figure 5). Amine oxidase activity in the MRV was abolished by the SSAO inhibitor, semicarbazide, and was reduced in the MPVAT, AF, and the SVF (Figures 6A–D). This is different from what was observed with tyramine as the substrate, where the reduction with semicarbazide was not significant. Tyramine is a poor substrate for SSAO compared to benzylamine with a K_m_ of 17.6 mM. Whereas, the K_m_ of benzylamine for SSAO is 161 μM (Precious and Lyles, 1988). Some of the tyramine-driven oxidase activity in the MRV could have been due to other amine oxidases other than SSAO. Oxidase activity in the brain was completely abolished by inhibition of MAO-B with pargyline, but was not affected by inhibition of SSAO (Figure 6E). Oxidase activity in the aorta was not affected by pargyline, but was reduced significantly by semicarbazide (Figure 6F).

### NE metabolism contributes to the anti-contractile effect of PVAT

**Figure 7:**
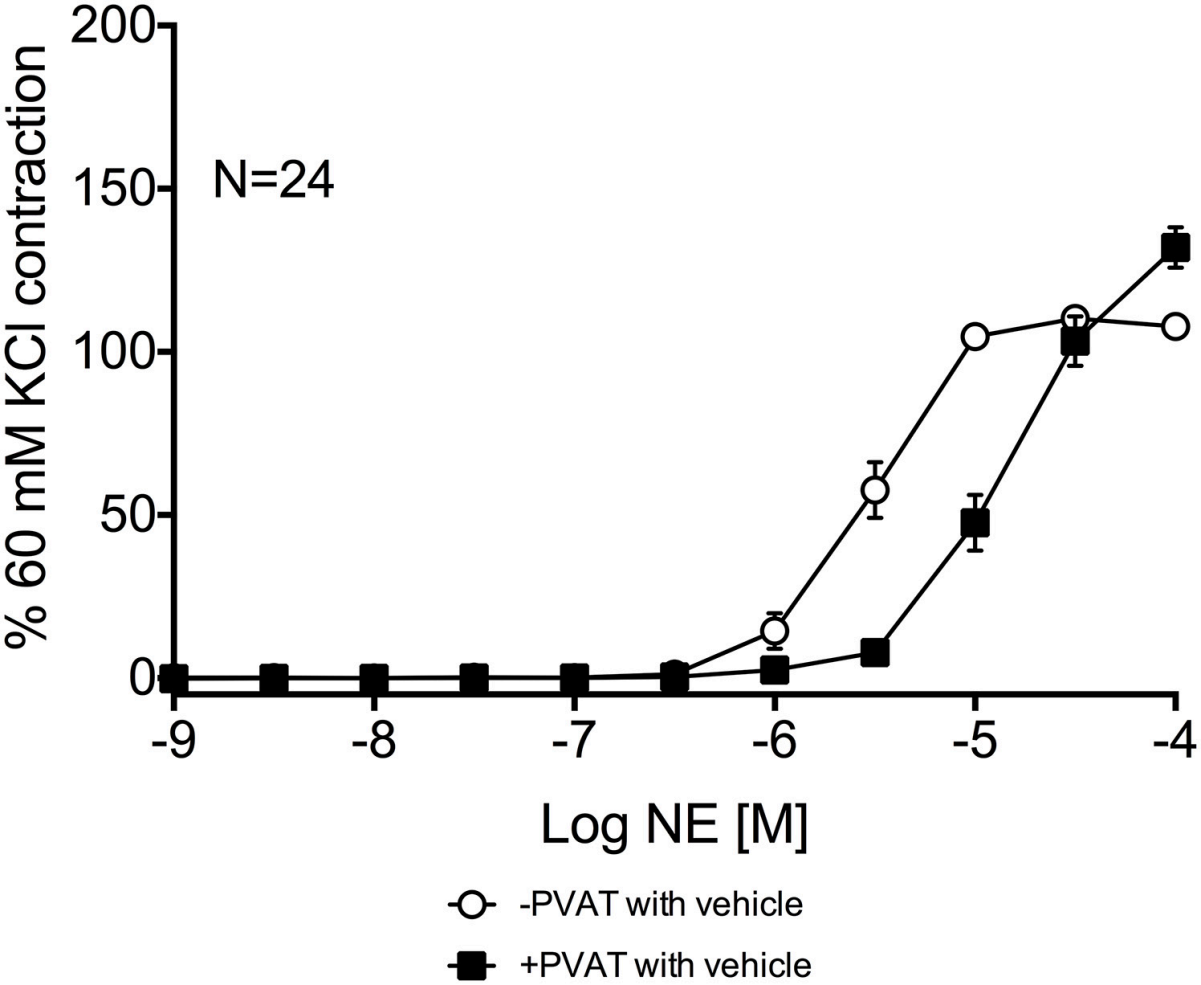
**PVAT exhibits an anti-contractile effect on mesenteric arteries exposed to NE**. NE-induced contraction of the rat mesenteric resistance arteries with or without PVAT and with vehicle (no inhibitors present). Bars represent means ± SEM. *N* = the number of animals used in each group.

**Figure 8:**
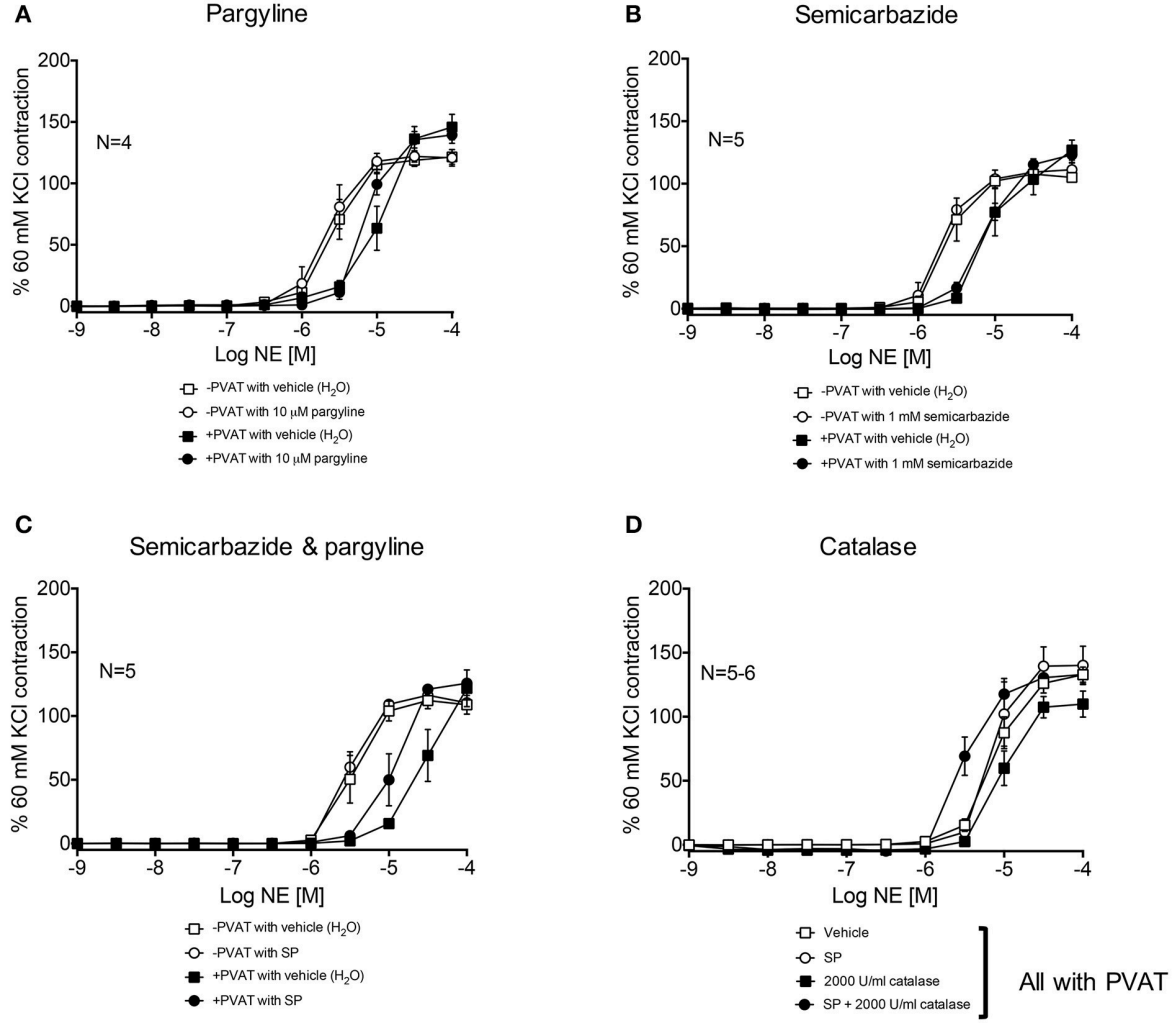
**Metabolism of NE and H_**2**_O_**2**_ release by PVAT inhibits contraction of rat mesenteric resistance arteries**. NE-induced contraction of the rat mesenteric resistance arteries with or without PVAT and with or without (vehicle) the following inhibitors of NE metabolism. **(A)** pargyline (10 μM; inhibits MAO-A and B), **(B)** semicarbazide (1 mM; inhibits SSAO), **(C)** SP = semicarbazide (1 mM) and pargyline (10 μM inhibits both MAO-A/B and SSAO). Arteries +PVAT were incubated with **(D)** SP and/or catalase (2000 U/ml). Force of contraction was normalized to the percent of the 60 mM KCl contraction. Bars represent means ± SEM. *N* = the number of animals used in each group.

Third-order mesenteric resistance arteries with (+) or without (−) PVAT were mounted on a 4-channel wire myograph. The force of contraction to 60 mM KCl of the arteries with and without PVAT were not different (Data Supplementary Figure 3). The arteries were incubated with either vehicle or an inhibitor prior to the generation of a NE concentration-response curve. The presence of PVAT gave a significant right-shift of the NE-response curve (Figure 7). NE had a −logEC_50_ [M] of 5.64 ± 0.05 in mesenteric resistance—PVAT and a −logEC_50_ [M] of 5.08 ± 0.06 in arteries +PVAT (Figure 7). Inhibition of MAO-A and B by 10 μM pargyline, or SSAO by semicarbazide (1 mM) did not shift the NE concentration-response curve of arteries + or −PVAT vs. vehicle (Figures 8A,B). However, a left-shift of the +PVAT curve was observed with incubation with semicarbazide + pargyline (10 μM; SP) suggesting that multiple NE metabolizers may contribute to PVAT's anti-contractile effect on mesenteric resistance arteries to NE (Figure 8C). To understand whether the shift in the NE-curve with SP was due to H_2_O_2_ being produced, we incubated +PVAT arteries with vehicle, SP, catalase (2000 U/ml), or SP + catalase (2000 U/ml) for 1 h prior to the addition of NE. The arteries that were incubated with SP and catalase had a left-shift in their response-curve to NE vs. those incubated with catalase only (Figure 8D). However, no shift was observed in the arteries that were exposed to SP vs. vehicle suggesting that inhibition of metabolism alone is not always sufficient to affect arterial response to NE and that both PVAT's metabolism of NE and production of H_2_O_2_ are involved in decreasing contraction to NE. The pharmacological parameters for Figures 8B–D are listed in Table 1.

**Table 1:** **Pharmacological parameters of isolated mesenteric resistance arteries with (+) or without (−) PVAT**.

**PVAT**	**Inhibitor**	**−LogEC_50_ (M)**	**Max Contraction %60 mM KCl**
−	Vehicle	5.74 ± 0.11	121.51 ± 5.98
−	10 μM pargyline	5.83 ± 0.12	120.82 ± 6.73
+	Vehicle	5.31 ± 0.15	145.90 ± 10.40
+	10 μM pargyline	5.40 ± 0.15	139.47 ± 6.84
−	Vehicle	5.66 ± 0.09	105.03 ± 3.57
−	1 mM semicarbazide	5.73 ± 0.08	111.17 ± 5.42
+	Vehicle	5.22 ± 0.11	126.92 ± 8.00
+	1 mM semicarbazide	5.29 ± 0.09	123.13 ± 6.05
−	Vehicle	5.55 ± 0.10	108.83 ± 7.34
−	SP	5.61 ± 0.09	110.35 ± 3.20
+	Vehicle	4.73 ± 0.11	121.78 ± 14.43
+	SP	5.13 ± 0.11	125.70 ± 2.56
+	Vehicle	5.36 ± 0.11	132.84 ± 5.90
+	SP	5.41 ± 0.16	142.81 ± 15.39
+	2000 U/ml catalase	5.10 ± 0.09	113.06 ± 9.12
+	SP + 2000 U/ml catalase	5.68 ± 0.13^a^	136.49 ± 8.37
−	Vehicle	5.72 ± 0.12	116.32 ± 0.57
−	100 μM corticosterone	5.99 ± 0.11	113.98 ± 9.26
+	Vehicle	4.98 ± 0.12	132.78 ± 18.89
+	100 μM corticosterone	5.14 ± 0.09	107.38 ± 3.39
−	Vehicle	5.71 ± 0.10	106.32 ± 3.33
−	1 μM nisoxetine	5.80 ± 0.10	103.55 ± 4.56
+	Vehicle	5.23 ± 0.15	141.99 ± 6.59
+	1 μM nisoxetine	5.92 ± 0.16^*^	134.94 ± 10.36
−	Vehicle	5.76 ± 0.15	131.05 ± 8.60
−	SPC	5.46 ± 0.12	88.31 ± 2.34^#^
+	Vehicle	5.27 ± 0.13	130.02 ± 6.19
+	SPC	5.41 ± 0.10	108.17 ± 3.63
−	Vehicle	5.57 ± 0.11	112.05 ± 6.07
−	SPN	5.81 ± 0.11	114.56 ± 3.15
+	Vehicle	4.66 ± 0.10	114.91 ± 3.44
+	SPN	5.86 ± 0.12^***^	120.69 ± 6.43

**Figure 9:**
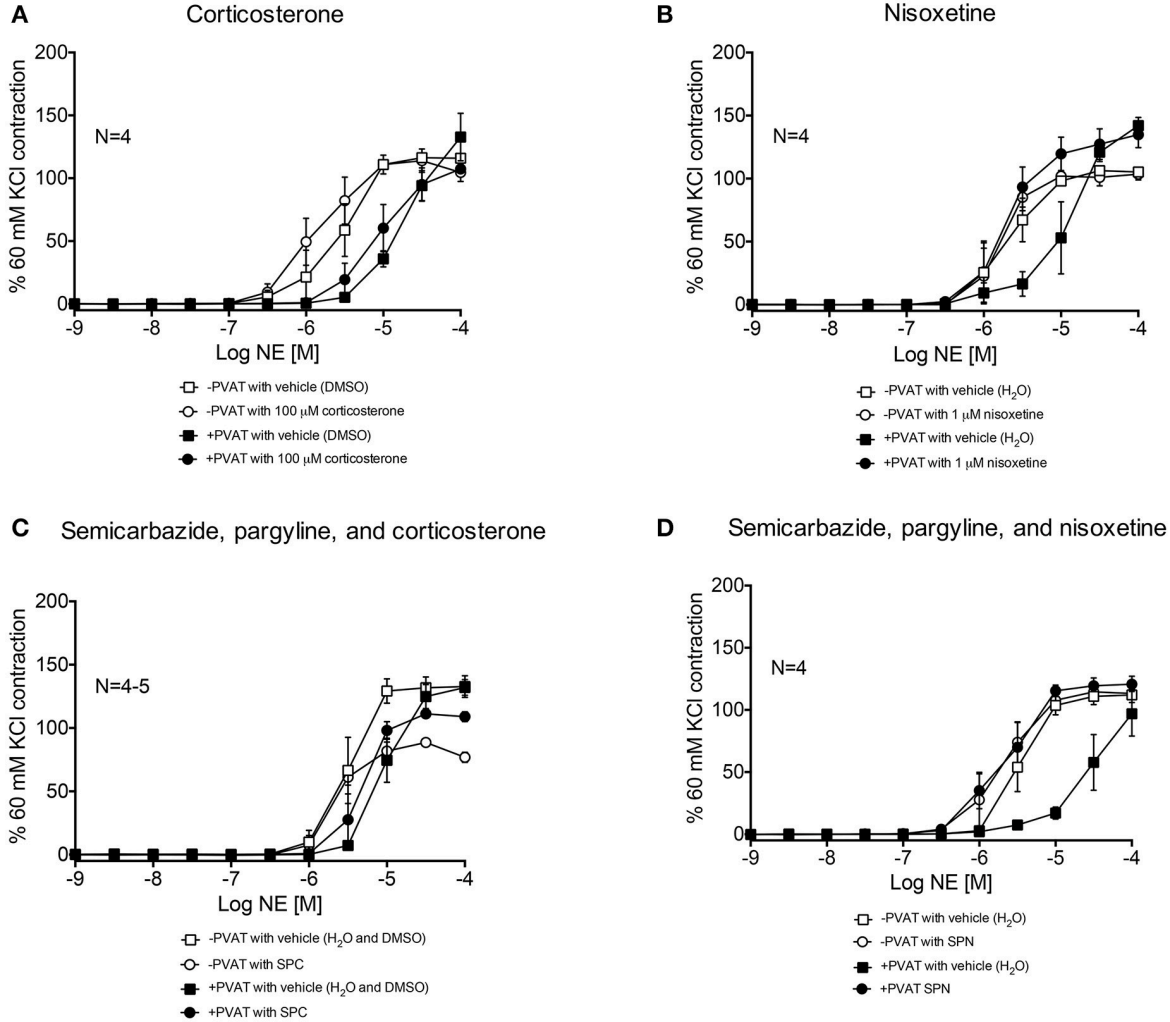
**NE-induced contraction of mesenteric resistance arteries with and without PVAT incubated with NE uptake inhibitors. (A)** NE-induced contraction of the rat mesenteric resistance arteries + or −PVAT, with corticosterone (100 μM; inhibits OCT3) or vehicle (H_2_O). **(B)** NE-induced contraction of the rat mesenteric resistance arteries + or −PVAT, incubated with nisoxetine (1 μM; inhibits NET) or vehicle (H_2_O). **(C)** SPC = semicarbazide (1 mM), pargyline (10 μM), and corticosterone (100 μM; used to inhibit SSAO, MAO-A/B and OCT3), and **(D)** SPN = semicarbazide (1 mM), pargyline (10 μM) and nisoxetine (1 μM; used to inhibit SSAO, MAO-A/B, and NET). The force of contraction was normalized to the percent of the 60 mM KCl contraction. Bars represent means ± SEM. *N* = the number of animals used in each group.

*Points represent mean ± SEM for values calculated from figures presented in Figures 8, 9. Unpaired Student's t-tests were performed to compare −logEC_50_ values between tissues with PVAT (+PVAT) and vehicle vs. +PVAT and inhibitor. Maximum contraction means were compared with a one-way ANOVA and a Holm's Sidak test for multiple comparisons. SPC = pargyline (10 μM), and corticosterone (100 μM). SPN = semicarbazide (1 mM), pargyline (10 μM) and nisoxetine (1 μM)*.

* *p < 0.05*,

*** *p < 0.001 vs. +PVAT with vehicle response*,

# *p < 0.05 vs. −/+ PVAT with vehicle*,

a *p < 0.05 vs. catalase (2000 U/ml)*.

### NE uptake contributes to the anti-contractile effect of PVAT

Arteries incubated with the organic cation transporter 3 (OCT3) inhibitor corticosterone (100 μM), had NE-response curves (+ and −PVAT) shifted to the left from vehicle (Figure 9A). However, the −logEC_50_ of the two curves were not statistically different (Table 1). Nisoxetine (1 μM), an inhibitor of the norepinephrine transporter (NET), shifted the NE-curve of +PVAT arteries significantly to the left vs. vehicle (Figure 9B; Table 1). We then tested whether transport of NE potentiated metabolism of NE in PVAT. Inhibition of SSAO [semicarbazide (1 mM)], MAO-A and B [pargyline (10 μM)], and OCT3 [corticosterone (100 μM); SPC] did not alter PVAT's anti-contractile effect when compared to vehicle (Figure 9C). The curve for the arteries +PVAT incubated with inhibitors was shifted slightly to the left from arteries +PVAT with vehicle but this shift was not significant (Table 1). It appears that inhibiting the entry of NE through OCT3 does not decrease NE removal. By contrast, inhibition of metabolism and uptake through NET by SPN [SPN = semicarbazide (1 mM), pargyline (10 μM), and nisoxetine (1 μM)] shifted the +PVAT curve to the left (Figure 9D) reflected by an increase in potency of NE on +PVAT arteries (Table 1).

## Discussion

PVAT contains releasable catecholamines and a system for uptake of NE (Ayala-Lopez et al., 2014, 2015). However, a mechanism for metabolism for amines in PVAT has not been investigated. This study demonstrates that the amine oxidase SSAO is present and highly active in rat MPVAT and MPVAT adipocytes. We also demonstrated that amine metabolism and NE uptake (which could assist in providing NE for metabolism intracellularly) contributes to the protective effect of PVAT.

Optimization of the Western protocol included the use of antibodies already validated or the validation of the antibodies. The MAO-A antibody was validated by our lab for its target specificity (Linder et al., 2008). The positive control bands on the Western blots for MAO-B were located by including a competing peptide in a validation experiment (Data Supplementary Figure 6). A Western blot was perform to select an appropriate positive control for COMT (Data Supplementary Figure 7). A competing peptide for the COMT antibody used was not commercially available. However, the Western data was consistent with low expression levels of mRNA detected. The appropriate band of interest for SSAO was located on the Western blot after including a competing peptide in a validation experiment (Data Supplementary Figure 8). Thus, we have confidence in the band identified by Western blot for SSAO.

Adipocytes in human abdominal and mammary white adipose tissue (WAT) contain both MAO-A and MAO-B (Pizzinat et al., 1999). Expression of mRNA and presence of protein for MAO-A was found in MPVAT and the MRV. However, expression of *Maob* was low in MPVAT. There was higher expression of *Aoc3* and SSAO in MPVAT vs. the MRV. SSAO is highly expressed in adipocytes and has several roles including adipogenesis (Mercier et al., 2001). In vascular smooth muscle cells, SSAO has roles in differentiation and the activation of glucose transport (El Hadri et al., 2002).

Pizzinat et al. (1999) suggested that the uptake-2 system (OCT3) in adipocytes would take up NE to be inactivated by MAO. We localized OCT3 to the PVAT adipocyte (Ayala-Lopez et al., 2015) and identified OCT3 as a mechanism for adipocytes to take up NE. Inhibition of NET also reduced NE uptake in MPVAT (Ayala-Lopez et al., 2015). Due to the presence of MAO-A and SSAO in MPVAT, we could expect metabolism of NE in PVAT to occur through transport-dependent and independent processes. If MAO-A is involved in the metabolism of amines in MPVAT, access to MAO would require an internalization of the amines from the extracellular environment and OCT3 and/or NET would be a way that NE can enter cells within MPVAT to be metabolized. SSAO is present on intracellular membranes (Enrique-Tarancón et al., 1998) and on the plasma membrane of adipocytes (Jalkanen and Salmi, 2001) where it is shed in a process that is regulated by TNF-alpha and insulin (Abella et al., 2004). Thus, entrance of catecholamines into adipocytes would not be required for SSAO action. However, inhibition of SSAO did not affect contraction of arteries to NE. Furthermore, inhibition of OCT3 and NE metabolism did not significantly shift the contractility curve but inhibiting NET alone or with SP shifted the curve of +PVAT arteries. Inactivation of NE by uptake through NET in MPVAT had the largest effect on contraction.

Stock and Westermann (1963) reported monoamine oxidase activity in rat epididymal adipose tissue. While, Bour et al. (2007) reported high MAO-A and SSAO activity in the AF from human subcutaneous adipose tissue. However, studies in PVAT had not been performed. We compared the amine oxidase activity from the MRV and the MPVAT to attain a whole picture of the oxidase activity in the vascular environment. SSAO was the predominant oxidase enzyme active in MPVAT. Surprisingly, we did not detect MAO-A activity in MPVAT although MAO-A protein and mRNA were present. The high SSAO activity could have masked lower monoamine oxidase activity and the MAO-A activity fell below our level of detection. The inclusion of the brain and aorta as controls assured us that the assay could detect and distinguish MAO and SSAO activity.

One limitation is that the substrates used in the oxidase activity assay, tyramine and benzylamine, are not the likely endogenous substrates for amine oxidases in PVAT. They were used as they are well characterized substrates for MAO and SSAO letting us come to more accurate conclusions as to which enzymes are active. Candidates for endogenous substrates for SSAO include aminoacetone, methylamine, 5-HT, and NE Lyles, 1995. Both NE and 5-HT are substrates of SSAO in rat brown adipose tissue (Barrand and Callingham, 1982). Unfortunately, NE could not be used in the oxidase assay because of its quick oxidization by the reagent in the Amplex Red assay (Elliott et al., 1989b; Zhao et al., 2012). The detection of NE metabolites after interaction with SSAO could assist in answering the question of whether NE is a substrate for SSAO in MPVAT. However, we did not perform these experiments as this assay is still in the development phase. Observed amine oxidase activities to NE and other prototypical substrates may be different when compared across species and adipose depots.

Another possibility is that degradation of amines by oxidation occurred by other enzymes not tested, especially in the MRV and the SVF where we were less successful at inhibiting the oxidase activity with the specific inhibitors used. For example, ceruloplasmin, an adipokine that is increased in the adipose tissue of obese humans (Arner et al., 2014), has broad specificity for degrading amines including NE, epinephrine, dihydroxyphenylalanine (DOPA), and 5-HT (Gutteridge and Stocks, 1981).

Due to the closeness of artery and vein in the mesenteric third order branches and the resulting inability to ascribe PVAT as belonging to either the artery and vein, both the artery and vein pair were used in the oxidase assays. The PVAT envelops both artery and vein, which are positioned close together and without a clear delineation of PVAT between the two. Thus, to gain an appreciation of the total tissue amine oxidases within the vascular environment we have kept the artery and vein together and used all of the PVAT that surrounds them for the Westerns and oxidase activity assays. In contractility, we have only used the artery since there is a larger literature base regarding PVAT's effect on arteries. However, more studies are needed to understand PVAT's effect on veins. Our group is highly interested in the veins of the splanchnic region and their role in peripheral resistance (Johnson et al., 2002; Ni et al., 2008; Watts et al., 2015; Seitz et al., 2016). Thus, PVAT's effect on veins is an avenue of future research.

Amine metabolism in rat mesenteric arteries has been found to occur mostly through SSAO, with less involvement from MAO-A (Elliott et al., 1989a). Elliott et al. (1989c) observed that inhibiting MAO or SSAO (with clorgyline or MDL 72145, respectively) did not shift the tyramine response curve of rat mesenteric arteries (without PVAT). However, a shift was achieved upon blocking both MAO and SSAO. In our studies, MAO and SSAO inhibition did not shift the NE-induced contraction in arteries −PVAT. By contrast, arteries +PVAT demonstrated a left-shift to NE when MAO, SSAO, and H_2_O_2_ were inhibited, supporting a redundant system for inactivating vasoactive amines that produces H_2_O_2._ H_2_O_2_ limits endothelial NO formation and reduces endothelium-dependent relaxation of the rat aorta (Sturza et al., 2013). In contrast, metabolism of 5-HT by MAO in the rat basilar artery was shown to lead to H_2_O_2_ generation, which by inhibiting the opening of BK_Ca2+_ channels, potentiated contraction (Poon et al., 2010). Our study supported that released H_2_O_2_ upon NE metabolism was contributing to the PVAT's anti-contractile effect.

**Figure 10:**
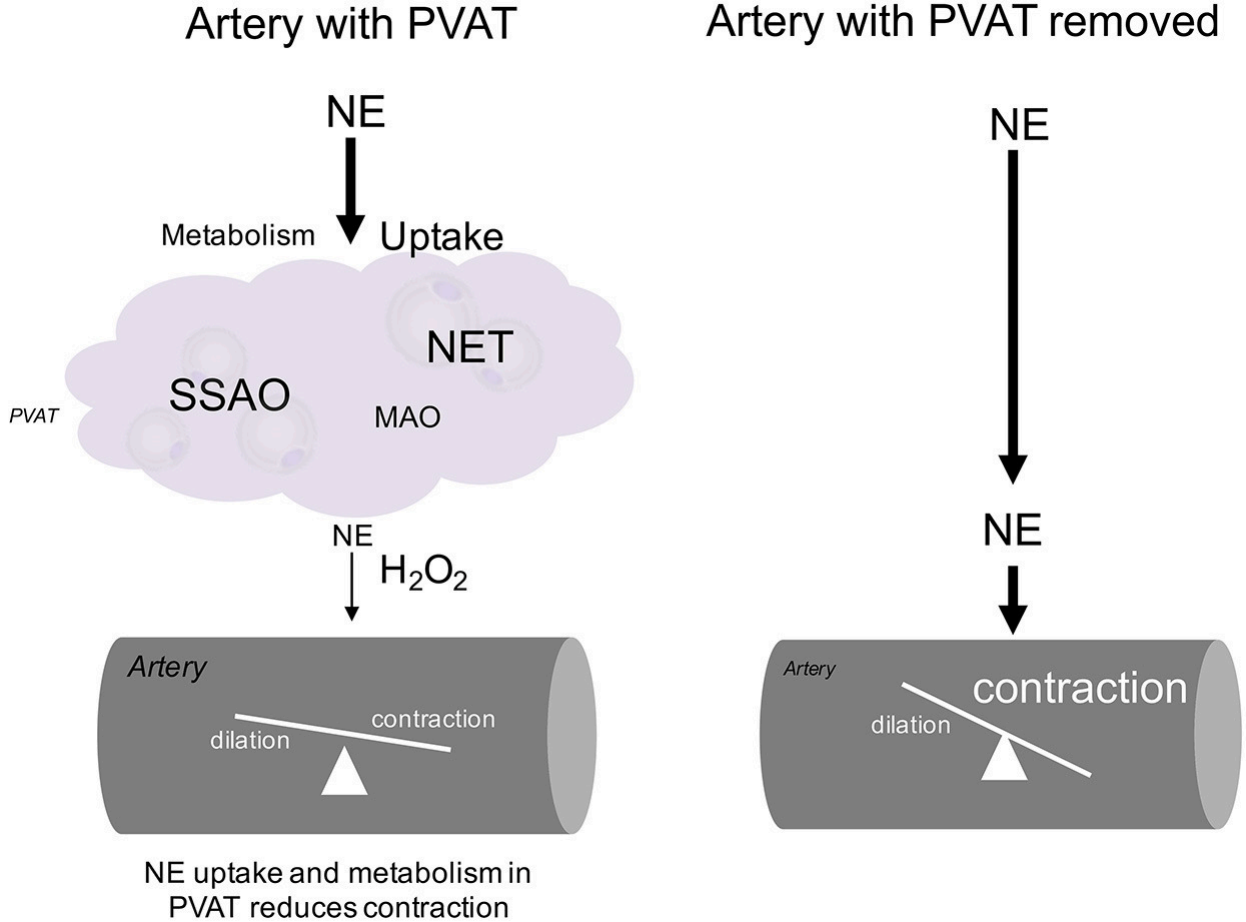
**Diagram of NE handling in PVAT**. The hypothesized mechanism for handling of NE by MPVAT and the MRV is shown above. PVAT contributes to the uptake and metabolism of NE from the adventitial side of the artery. Without PVAT, more NE reaches the artery to cause contraction.

Our proposed hypothesis is illustrated in Figure 10. On the left, an artery with PVAT is exposed to NE. The NE is taken up through NET and subsequently is metabolized in the PVAT either through SSAO or MAO. A small amount of NE reaches the artery relative to the amount of NE originally released along with H_2_O_2_ and less contraction of the artery occurs. On the right, an artery with the PVAT removed is exposed to NE. The full amount of NE reaches the artery to cause contraction. A small amount of NE (not enough to affect contraction) may be metabolized within the artery.

We can place our findings in context of the disease that brought us to examine amine metabolism in PVAT. SSAO activity in MPVAT could influence vascular inflammation as it has a role in inflammation and is being explored as a biomarker for atherosclerosis (Mészáros et al., 1999; Karadi et al., 2002). In obesity, SSAO and MAO activity near the vessel could promote vascular remodeling. We do not have data to support this. Thus, investigation into SSAO and MAO activity in PVAT during obesity in necessary.

## Conclusions

The MRV and MPVAT with its corresponding AF and SVF contain amine oxidase activity mostly attributed to SSAO. Amine metabolism and NE uptake in PVAT protects arteries from contraction to NE. Studies are necessary to understand how this NE handling system functions in human PVAT and how it may be altered in obesity.

## Author contributions

NA: Conceived the study, designed and executed the experiments, analyzed and interpreted the data, wrote and revised the manuscript. JT: Performed the Western blots and assisted in writing the methods for the Western experiments, read and revised the manuscript. SW: Conceived the study, guided the experimental design, data analysis and interpretation, read and revised the manuscript.

## Funding

This work was funded by NIH NHLBI P01HL70687 and F31 HL128035.

### Conflict of interest statement

The authors declare that the research was conducted in the absence of any commercial or financial relationships that could be construed as a potential conflict of interest.

## Supplementary material

Data Supplementary Figure 1 **Image representing the rat mesenteric vessels and PVAT used in this study**. The location of the mesenteric resistance artery-vein pair used is indicated by the red arrow and their associated MPVAT is indicated by the white arrow.

Data Supplementary Figure 2 **Image representing the AF and the SVF isolated from whole MPVAT**. Representative of the following experiments where the AF (left) and SVF (right) were used. Phase contrast images were taken with a 20 × objective. The black scale bar = 100 μM.

Data Supplementary Figure 3 **Comparison of the force of contraction between the +PVAT and −PVAT mesenteric resistance arteries**. Arteries were exposed to 60 mM KCl at the beginning of each experiment. Graphed are the “initial” force of contraction of the arteries + and −PVAT from the experiments shown in Figures 8, 9. Data were compared with a paired *t*-test and shown to be non-significant (*p* = 0.20). Bars represent means ± SEM. *N*, the number of animals used in each group. NS, non-significant (*p* > 0.05).

Data Supplementary Figure 4 **Comparison of the force of contraction between the +PVAT and −PVAT mesenteric resistance arteries at the initial part of the experiment vs. the end of experiment**. Arteries were exposed to 60 mM KCl at the beginning of each experiment and at the end. Graphed are the “initial” vs. the “end” forces of contraction of the arteries + and −PVAT from Figure 8. Data were compared with a 2-way ANOVA and shown to be non-significant. Bars represent means ± SEM. *N*, the number of animals used in each group.

Data Supplementary Figure 5 **Comparison of the force of contraction between the +PVAT and −PVAT mesenteric resistance arteries at the initial part of the experiment vs. the end of experiment**. Arteries were exposed to 60 mM KCl at the beginning of each experiment and at the end. Graphed are the “initial” vs. the “end” forces of contraction of the arteries + and −PVAT from Figure 9. Data were compared with a 2-way ANOVA. Bars represent means ± SEM. *N*, the number of animals used in each group. ^*^*p* < 0.05.

Data Supplementary Figure 6 **MAO-B Western blot optimization experiment**. Western blots for MAO-B were prepared that were exposed to anti-MAO-B antibody (no peptide; left) or the antibody and the competing peptide (right) to locate the band for MAO-B and select the appropriate positive control tissue. MAO-B signal was observed at around 45 kDa. The stomach fundus gave the least non-specific signal and thus was selected as the positive control.

Data Supplementary Figure 7 **COMT Western blot optimization experiment**. Western blots for COMT were prepared to select the proper positive control tissue. COMT signal was observed at around 45 kDa. The competing peptide was not commercially available. The Jurkat whole cell lysate gave the least non-specific signal and thus was selected as the positive control.

Data Supplementary Figure 8 **VAP-1 (SSAO) Western blot optimization experiment**. Western blots for VAP-1 (SSAO) were prepared that were exposed to anti-VAP-1 antibody (no peptide; left) or the antibody and the competing peptide (right) to locate the band for VAP-1. VAP-1 signal was observed at around 80 kDa. The lung was the positive control in this experiment. However, because the signal was not strong, the aorta was used as the positive control in subsequent experiments.

## Abbreviations

5-HT: 5-hydroxytryptamine
AF: adipocyte fraction
COMT: catechol-o-methyl-transferase
DMSO: dimethyl sulfoxide
MAO: monoamine oxidase
MPVAT: mesenteric perivascular adipose tissue
MRV: mesenteric resistance vessels
NE: norepinephrine
NET: norepinephrine transporter
OCT3: organic cation transporter 3
PVAT: perivascular adipose tissue
SVF: stromal vascular fraction
SSAO: semicarbazide sensitive amine oxidase
WAT: white adipose tissue.
