# Supplementary figures and images for: Perivascular Adipose Tissue's Impact on Norepinephrine-Induced Contraction of Mesenteric Resistance Arteries
Source: Front Physiol. 2017 Feb 8;8:37. doi: 10.3389/fphys.2017.00037 (PMC5296360; doi:10.3389/fphys.2017.00037)

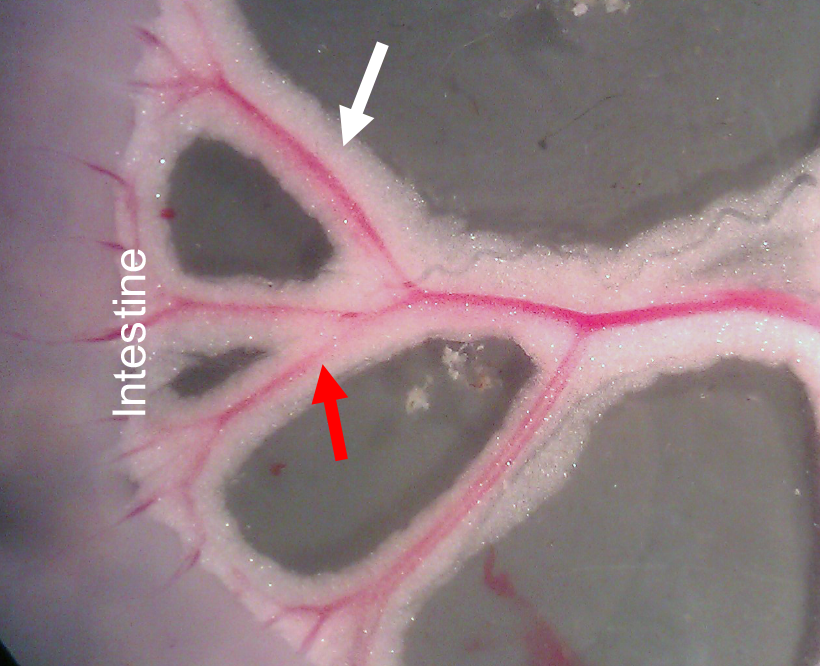

Supplement: Data Supplementary Figure 1 — Image representing the rat mesenteric vessels and PVAT used in this study. The location of the mesenteric resistance artery-vein pair used is indicated by the red arrow and their associated MPVAT is indicated by the white arrow. [file Image1.TIF]

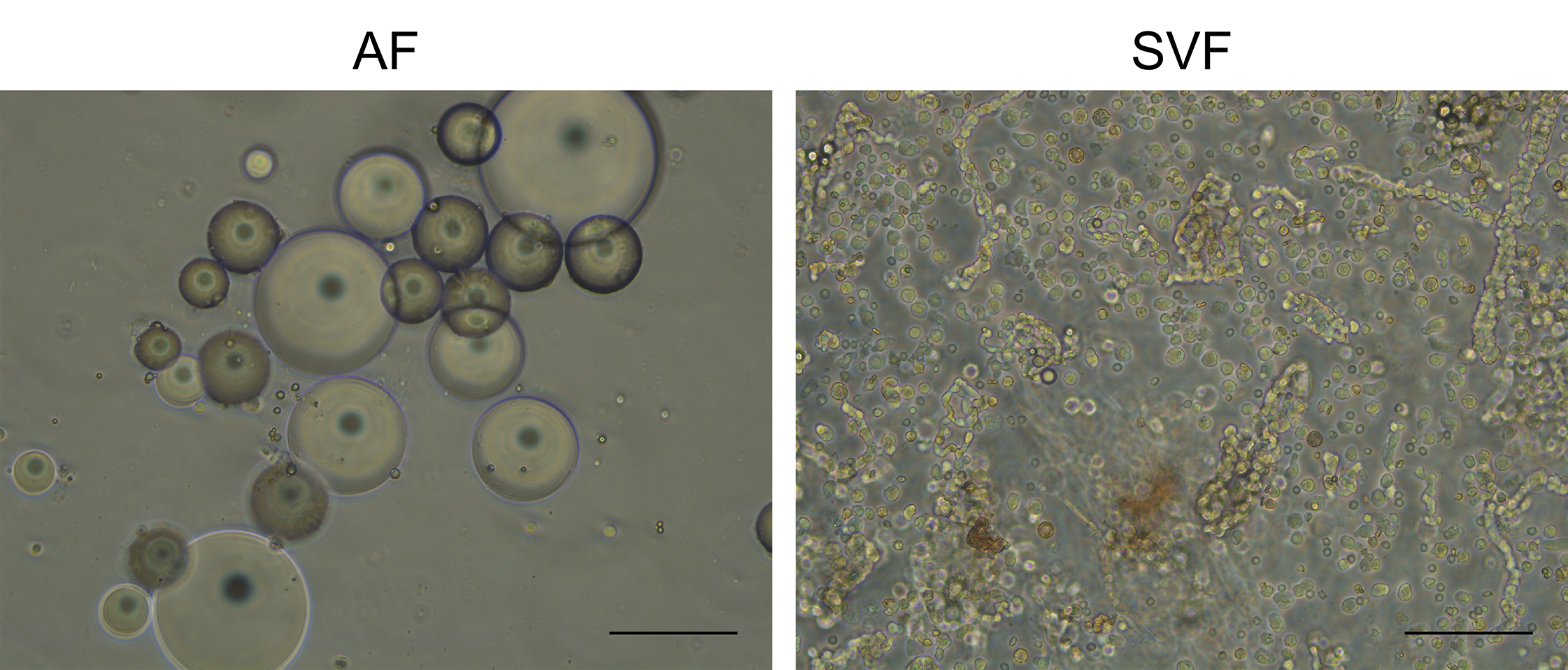

Supplement: Data Supplementary Figure 2 — Image representing the AF and the SVF isolated from whole MPVAT. Representative of the following experiments where the AF (left) and SVF (right) were used. Phase contrast images were taken with a 20 × objective. The black scale bar = 100 μM. [file Image2.tif]

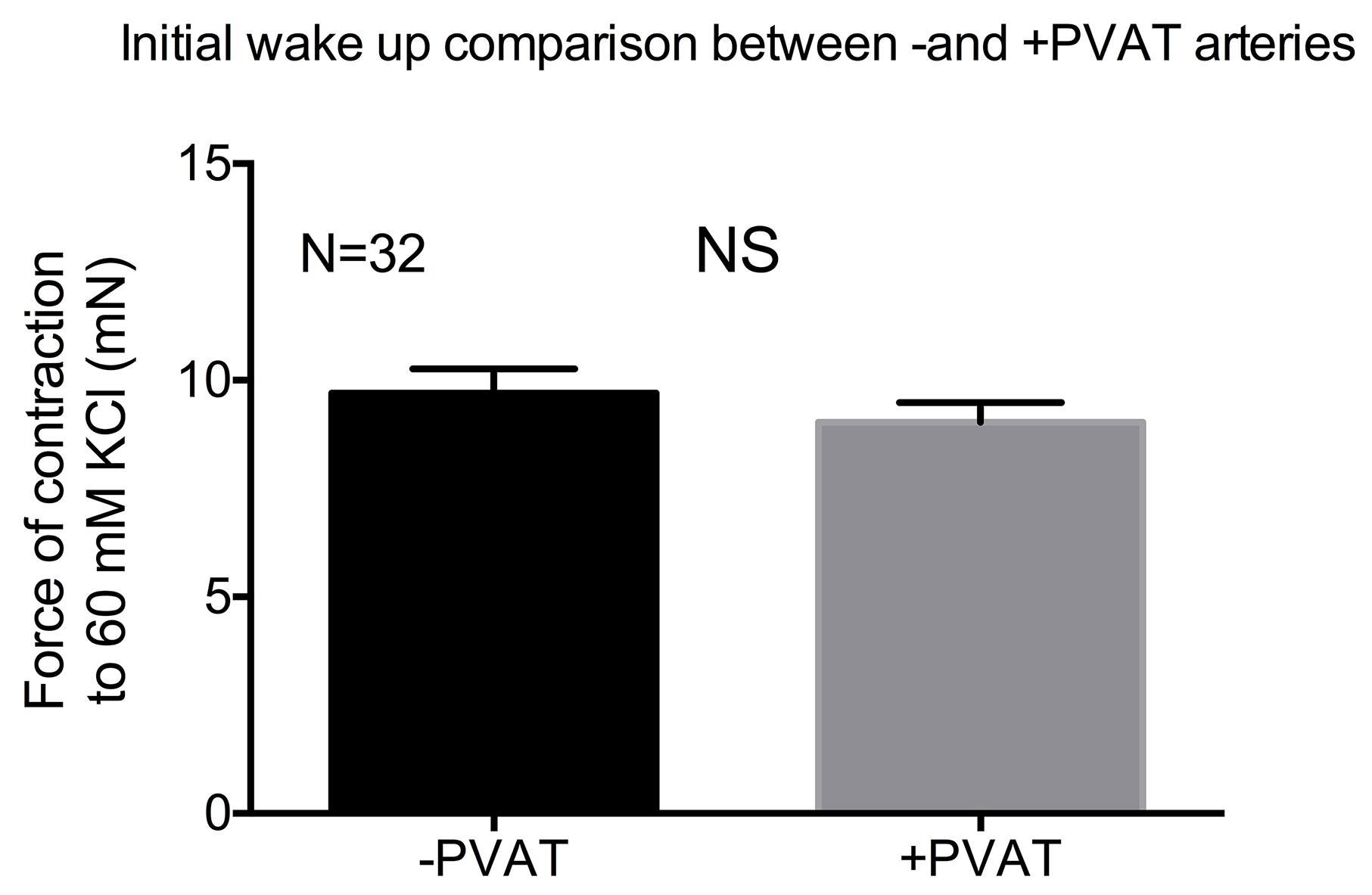

Supplement: Data Supplementary Figure 3 — Comparison of the force of contraction between the +PVAT and −PVAT mesenteric resistance arteries. Arteries were exposed to 60 mM KCl at the beginning of each experiment. Graphed are the “initial” force of contraction of the arteries + and −PVAT from the experiments shown in Figures 8, 9. Data were compared with a paired t-test and shown to be non-significant (p = 0.20). Bars represent means ± SEM. N, the number of animals used in each group. NS, non-significant (p > 0.05). [file Image3.tif]

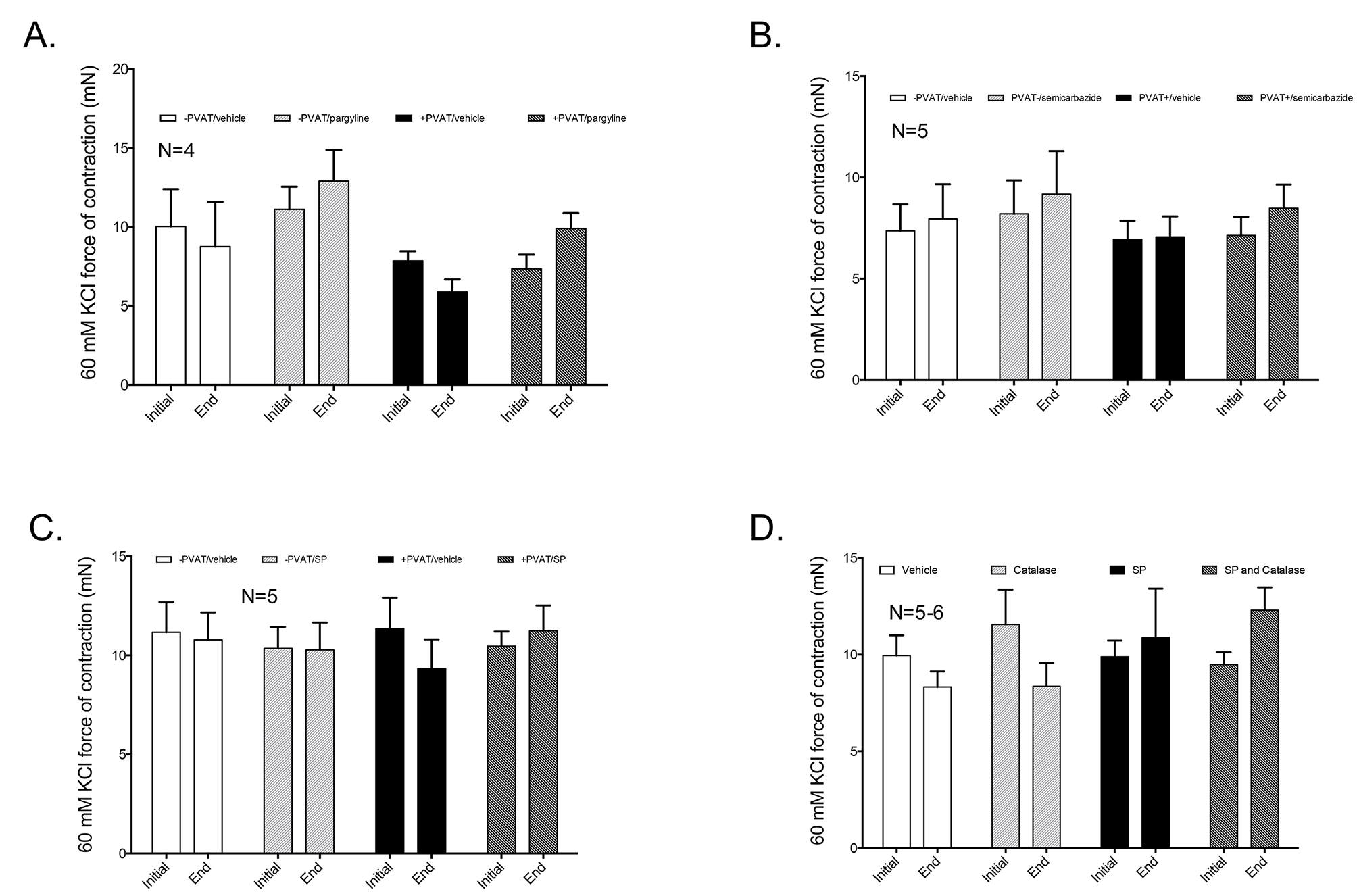

Supplement: Data Supplementary Figure 4 — Comparison of the force of contraction between the +PVAT and −PVAT mesenteric resistance arteries at the initial part of the experiment vs. the end of experiment. Arteries were exposed to 60 mM KCl at the beginning of each experiment and at the end. Graphed are the “initial” vs. the “end” forces of contraction of the arteries + and −PVAT from Figure 8. Data were compared with a 2-way ANOVA and shown to be non-significant. Bars represent means ± SEM. N, the number of animals used in each group. [file Image4.tif]

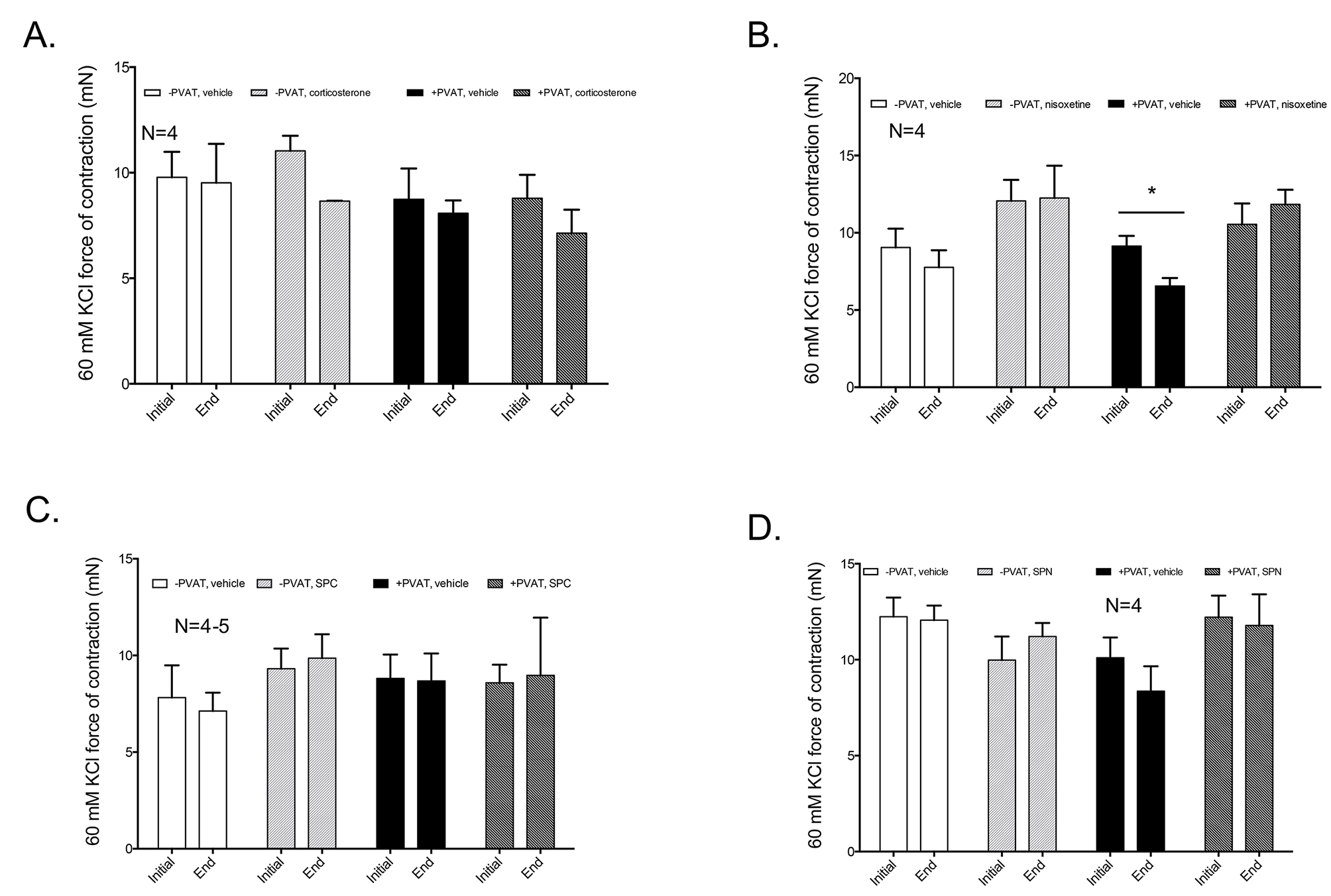

Supplement: Data Supplementary Figure 5 — Comparison of the force of contraction between the +PVAT and −PVAT mesenteric resistance arteries at the initial part of the experiment vs. the end of experiment. Arteries were exposed to 60 mM KCl at the beginning of each experiment and at the end. Graphed are the “initial” vs. the “end” forces of contraction of the arteries + and −PVAT from Figure 9. Data were compared with a 2-way ANOVA. Bars represent means ± SEM. N, the number of animals used in each group. *p < 0.05. [file Image5.tif]

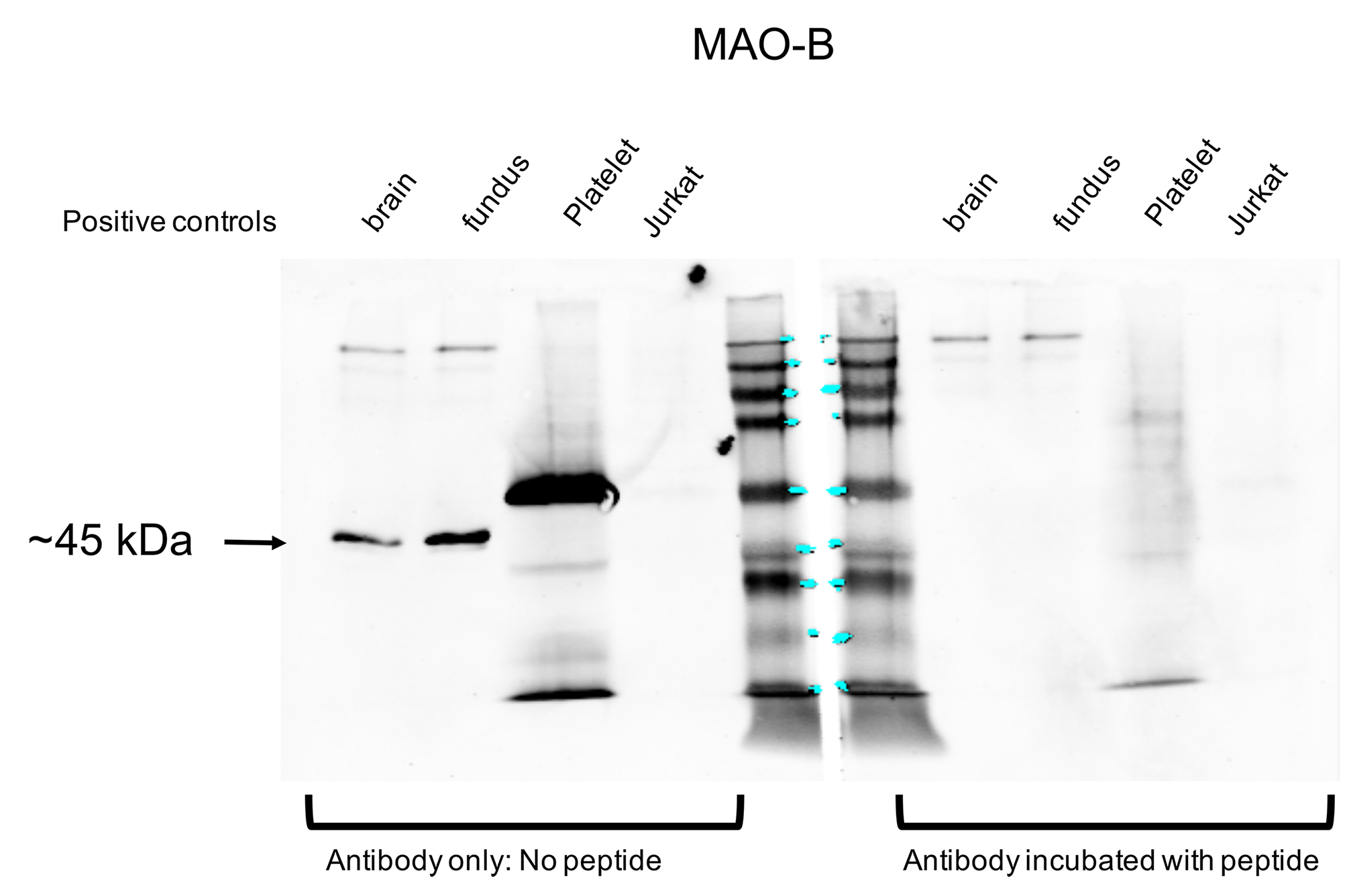

Supplement: Data Supplementary Figure 6 — MAO-B Western blot optimization experiment. Western blots for MAO-B were prepared that were exposed to anti-MAO-B antibody (no peptide; left) or the antibody and the competing peptide (right) to locate the band for MAO-B and select the appropriate positive control tissue. MAO-B signal was observed at around 45 kDa. The stomach fundus gave the least non-specific signal and thus was selected as the positive control. [file Image6.tif]

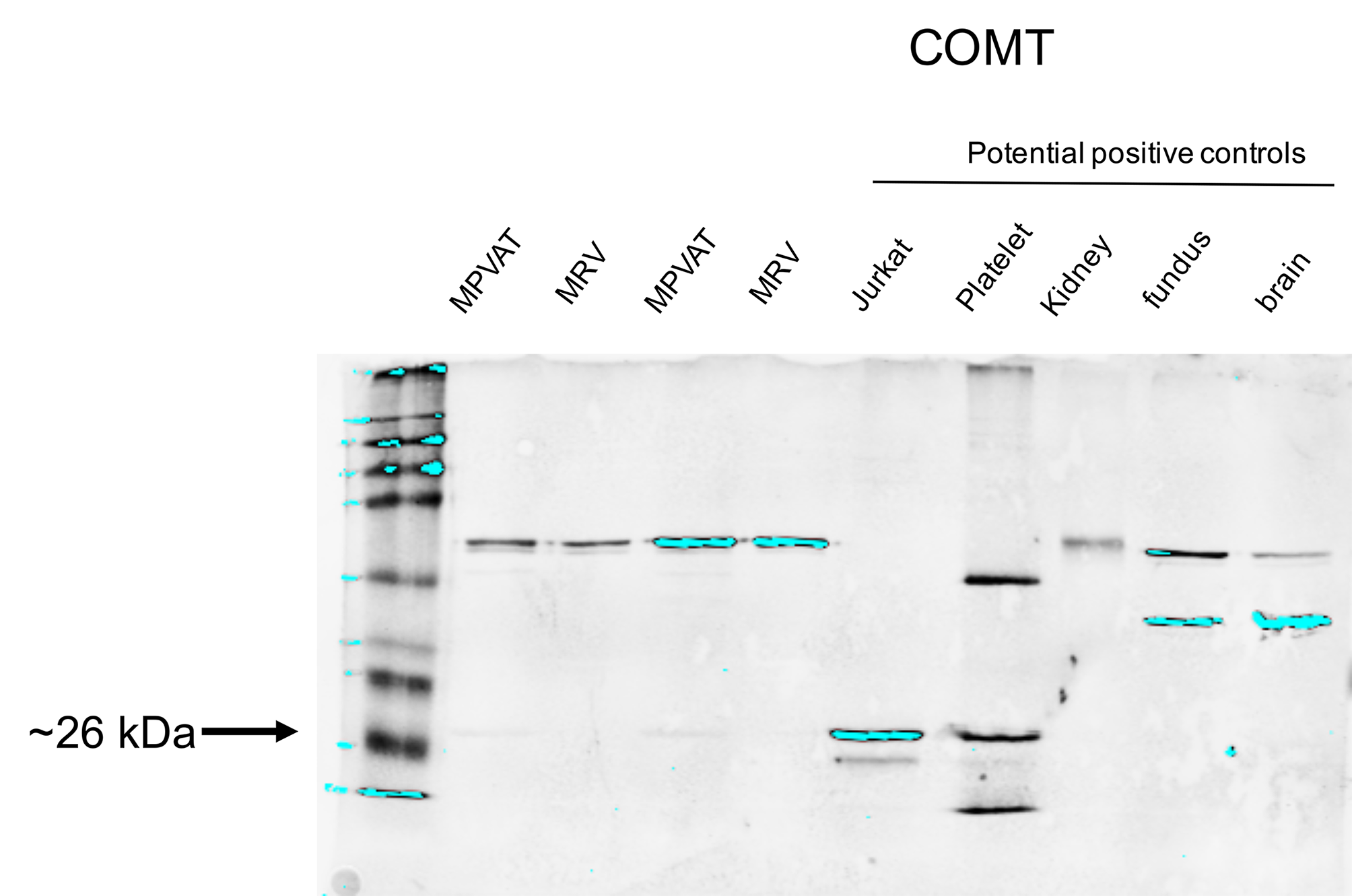

Supplement: Data Supplementary Figure 7 — COMT Western blot optimization experiment. Western blots for COMT were prepared to select the proper positive control tissue. COMT signal was observed at around 45 kDa. The competing peptide was not commercially available. The Jurkat whole cell lysate gave the least non-specific signal and thus was selected as the positive control. [file Image7.tif]

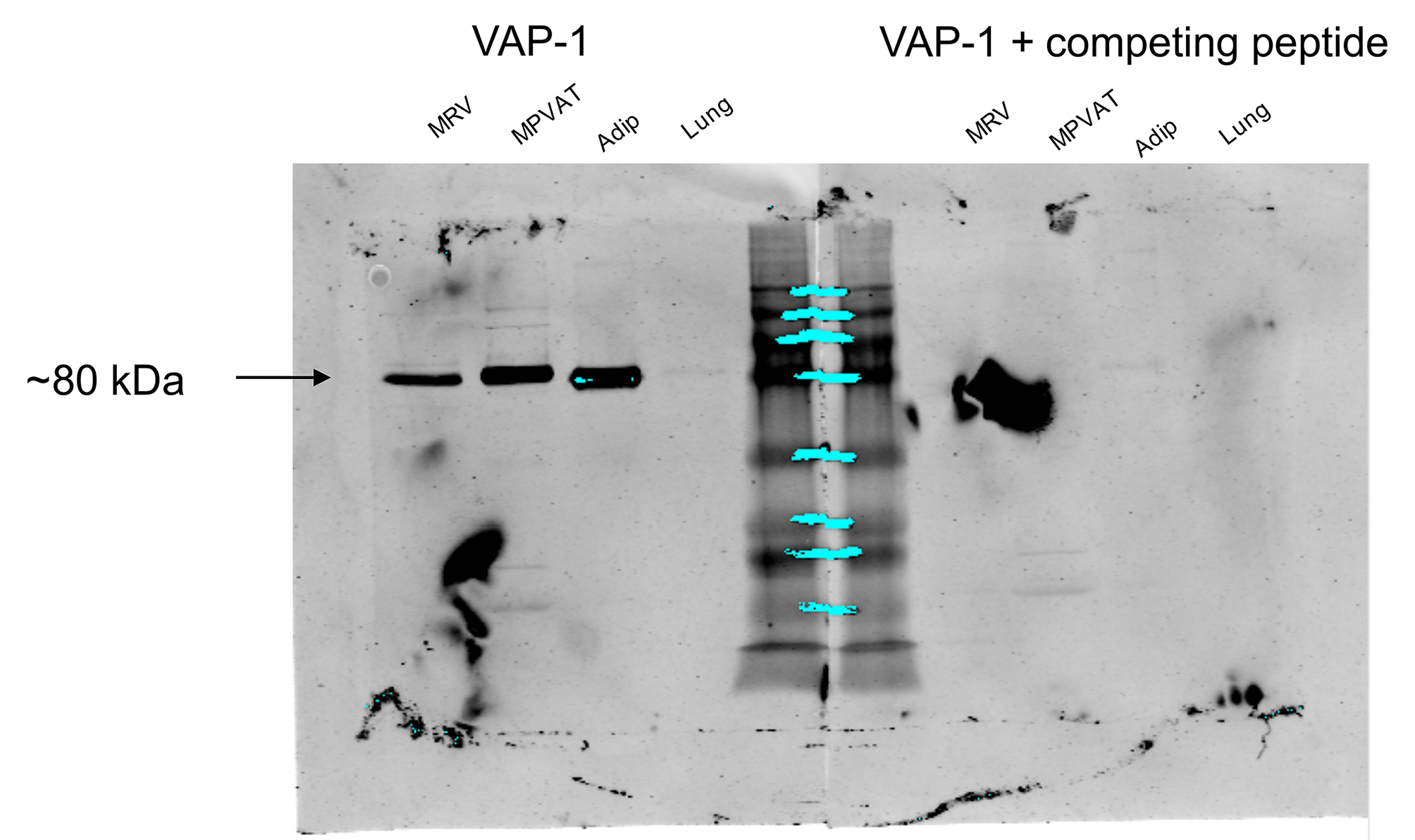

Supplement: Data Supplementary Figure 8 — VAP-1 (SSAO) Western blot optimization experiment. Western blots for VAP-1 (SSAO) were prepared that were exposed to anti-VAP-1 antibody (no peptide; left) or the antibody and the competing peptide (right) to locate the band for VAP-1. VAP-1 signal was observed at around 80 kDa. The lung was the positive control in this experiment. However, because the signal was not strong, the aorta was used as the positive control in subsequent experiments. [file Image8.tif]
